# The Potential of Cyclodextrins as Inhibitors for the BM2 Protein: An In Silico Investigation

**DOI:** 10.3390/molecules29030620

**Published:** 2024-01-28

**Authors:** Aijun Liu, Hao Zhang, Qingchuan Zheng, Song Wang

**Affiliations:** 1 Institute of Theoretical Chemistry, College of Chemistry, Jilin University, Changchun 130023, China; liuaj20@mails.jlu.edu.cn (A.L.); stringbell@jlu.edu.cn (H.Z.); 2 School of Pharmaceutical Sciences, Jilin University, Changchun, 130021, China

**Keywords:** influenza B virus, BM2 proton channel, cyclodextrins, inhibitor, molecular dynamics

## Abstract

The influenza BM2 transmembrane domain (*BM2_TM_*), an acid-activated proton channel, is an attractive antiviral target due to its essential roles during influenza virus replication, whereas no effective inhibitors have been reported for BM2. In this study, we draw inspiration from the properties of cyclodextrins (CDs) and hypothesize that CDs of appropriate sizes may possess the potential to act as inhibitors of the ${BM2}_{TM}$ proton channel. To explore this possibility, molecular dynamics simulations were employed to assess their inhibitory capabilities. Our findings reveal that CD4, CD5, and CD6 are capable of binding to the ${BM2}_{TM}$ proton channel, resulting in disrupted water networks and reduced hydrogen bond occupancy between H19 and the solvent within the ${BM2}_{TM}$ channel necessary for proton conduction. Notably, CD4 completely obstructs the ${BM2}_{TM}$ water channel. Based on these observations, we propose that CD4, CD5, and CD6 individually contribute to diminishing the proton transfer efficiency of the BM2 protein, and CD4 demonstrates promising potential as an inhibitor for the BM2 proton channel.

## 1. Introduction

Influenza B virus infections, representing 23% of reported influenza cases, pose a significant public health threat, with a notable 52% fatality rate in pediatric cases [1,2,3,4]. The constant genetic drift of the virus increases the risk of rendering existing vaccines and anti-influenza drugs ineffective [4,5,6,7]. Consequently, there is an urgent need to design and develop novel antiviral drugs specifically tailored to target the influenza B virus.

The Influenza B virus matrix protein 2 (BM2), situated on the virus envelope, forms a homotetramer that functions as a proton-selective channel [8]. Upon entering host cells through receptor-mediated endocytosis, the BM2 channel activates in the low pH environment of the endosome, facilitating proton entry into the virus interior [9,10,11]. This acidification separates the ribonucleoprotein (RNP) from the matrix protein 1 (BM1), allowing it to enter the host cell for transcription and replication, completing the viral lifecycle.

**Figure 1:**
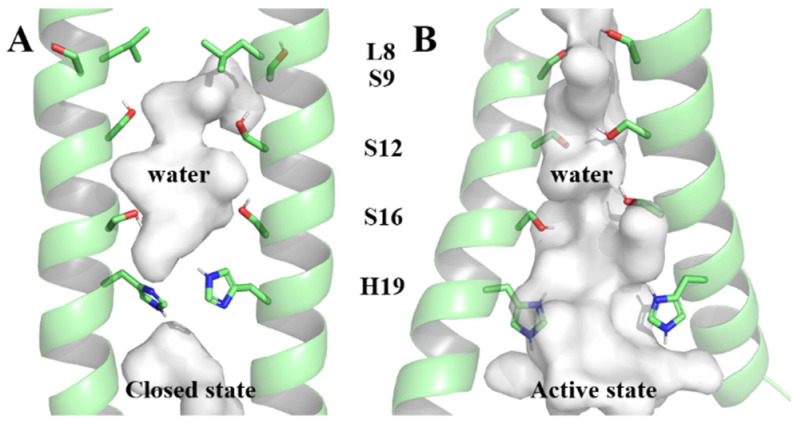
The closed state and active state of the ${BM2}_{TM}$ channel within pore-lining residues serine triplet and H19. (**A**) The closed state of the ${BM2}_{TM}$ channel, (**B**) The active state of the ${BM2}_{TM}$ channel.

Comprising 109 residues, each BM2 monomer includes a transmembrane region (${BM2}_{TM}$) responsible for proton transfer [8,12,13,14]. Two ${BM2}_{TM}$ structures (6PVR and 6PVT), representing closed and open states under physiological and acidic conditions, were captured in the lipid bilayer by the Hong Lab using solid-state nuclear magnetic resonance (ss-NMR) [15]. In the open state, the transmembrane helices of ${BM2}_{TM}$ are more tilted and more separated from each other [15], resulting in a larger water-filled pore and facilitating for proton conduction [16,17]. The residue L8, as a bottleneck of the ${BM2}_{TM}$ channel under physiological pH, will conveniently open under acidic conditions for water molecules to pass through [15]. The inner surface of the ${BM2}_{TM}$ channel is occupied by polar serine triplet (Serine9, Serine12, and Serine16 abbreviated in S9, S12, and S16, respectively) and titrable Histidine19 (H19, all residues mentioned in the following text are represented by the abbreviation and corresponding sequence number of the residues at the respective positions on the BM2 protein) [15,18,19] (as shown in Figure 1). The serine triplet coordinates water molecules [19,20], forming continuous hydrogen-bonding networks that facilitate proton conduction [15,21]. The tetramer H19, which expands in diameter by 1.4 Å at low pH [15], is responsible for shuttling protons through the formation of hydrogen bonds with water molecules [16,21,22], aligning with the mechanics of proton transfer to, and away from, the proton-selective H37 in the influenza A virus matrix protein 2 (AM2) [23,24,25,26].

Due to the essential role in the influenza B virus lifecycle, the BM2 channel emerges as an attractive antiviral target [9,10,11,14,27]. Blocking proton transfer within the BM2 channel can prevent the acidification of the virus interior, inhibit viral uncoating, and ultimately suppress viral replication. Amantadine and rimantadine, recognized as this type of inhibitors of AM2, a functional analog of BM2 [28,29,30], are proven ineffective against BM2 protein [8,12,31,32]. Lamb et al. proposed that the presence of polar serine residues within the BM2 channel might render adamantanes ineffective [14]—a perspective subsequently supported by multiple studies [17,18,19]. Hong et al. suggest that the polar pore-lining serine residues disfavor the binding of nonpolar drugs, underscoring the importance of targeting the polar pore-lining surface of the BM2 channel [17]. Recently, our laboratory’s molecular dynamics simulations revealed spatial geometric mismatch as another critical factor contributing to adamantanes’ ineffectiveness in inhibiting proton transfer within the BM2 channel [33] Despite being an attractive antiviral target, no inhibitors have been reported for the BM2 protein.

Identifying compounds capable of binding to the hydrophilic inner surface of the BM2 channel [17] and complementing the geometric configuration of the BM2 channel cavity [33] to disrupt the continuous hydrogen-bonding water networks necessary for proton conduction [22,34,35], may represent an effective strategy for inhibitor development. Cyclodextrin molecules (CDs) align seamlessly with this approach. Composed of α-D-glucopyranose units, CDs exhibit a toroidal structure with hydrophilic outer edges and a hydrophobic interior and are available in a variety sizes [36,37,38,39]. The hydrophilic outer edge of CDs may have the potential to bind to the hydrophilic inner surface of the ${BM2}_{TM}$ channel, while the smaller hydrophobic interior may disrupt the continuous hydrogen-bonding water networks within the BM2 channel. Moreover, previous studies have employed CDs as well as derivatives to inhibit bacterial ion channel proteins, such as α-hemolysin (α-HL) of *Staphylococcus aureus* and the protective antigen (PA) component of anthrax exotoxin (PA63 protein), both forming heptameric transmembrane pores with ion transport capabilities [40,41,42,43]. Although the mechanisms of action of α-HL, anthrax toxin, and BM2 are different [8,13,14,44,45,46,47,48], the common feature is the formation of oligomeric transmembrane pores with ion conductance capabilities [8,14,40,41]. Additionally, the polar cyclodextrin molecules can be capsulated into nanoliposomes making them more easily to cross various membranes to reach the interior of the endosome [49,50,51,52,53].

Based on the structural characteristics of CDs [36,37,38,39] and these application precedents [40,41,42,43], we hypothesize that appropriately sized CDs may show promise in inhibiting the BM2 proton channel. To substantiate this view, we employed a combined approach of molecular docking and molecular dynamics simulations to assess the potential of appropriately sized CDs (CD4, CD5, and CD6) as inhibitors for the BM2 protein and reveal possible mechanisms of inhibition. The use of molecular dynamics simulation methods allows for a more cost-effective and efficient exploration of the interaction between inhibitors and the target protein at an atomic resolution level, which is challenging to accomplish through experimental means [33,34,35]. Therefore, it is suitable for the study of inhibitors for this type of system. This work would yield valuable insights into the potential mechanisms through which CDs might impact the functionality of the BM2 channel protein, providing essential guidance for future drug development endeavors and establishing a solid groundwork for the design of highly effective BM2 channel protein inhibitors.

## 2. Results and Discussion

### 2.1. Selection of the BM2 Protein and the CDs Structures

The transmembrane part (1–33) of BM2 protein-${BM2}_{TM}$, is sufficient to mediate proton-selective conductance, and its specific conductance is indistinguishable from that of the full-length BM2 protein [13]. Therefore, our study will only focus on the transmembrane domain of the BM2 protein. The NMR structures of the ${BM2}_{TM}$ available in the Protein Data Bank (PDB) include 2KIX [19], 6PVR, and 6PVT [15]. Among these, the lowest energy structures of the 6PVR and 6PVT ensemble were chosen for this study due to their higher resolution and relevance to membrane-like environments [15]. The structures were processed for protonation using the online server PDB2PQR [54]. The resulting models included a protonation state of 0/0 for the H19/H27 tetramer in 6PVR and +4/+4 for the H19/H27 tetramer in 6PVT [17,21].

**Figure 2:**
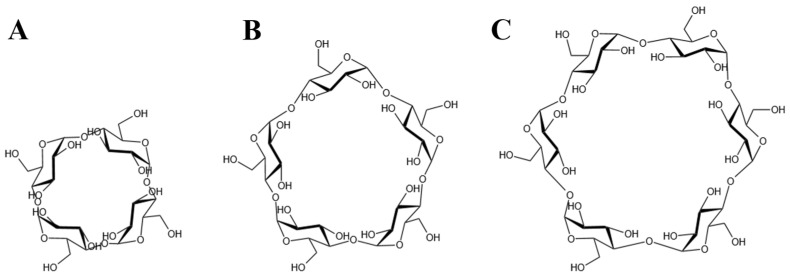
The chemical formula of CDs. (**A**) The CD4 structure, (**B**) The CD5 structure and (**C**) The CD6 structure.

Considering the maximum aperture of the ${BM2}_{TM}$ is approximately 14 Å [15], CDs larger than CD6, which has an outer diameter of 13.7 Å, were deemed incompatible with the channel space. Therefore, CD4 [55], CD5 [56], and CD6 [57] were selected for subsequent investigations (Figure 2).

The molecular structure of CD6 used in simulations was obtained from the Protein Data Bank [58]. The CD5 and CD4 structures were constructed by removing one and two α-D-glucopyranose units, respectively, from CD6 using GaussView 6 [59]. Structural optimization of CD4, CD5, and CD6 was performed using the Gaussian 16 software [60] under B3LYP/6-31G basis set [61,62]. The optimized structures (the partial charges of each atom were appended in the Supplementary Materials) were utilized in the construction of subsequent complex models.

### 2.2. Molecular Docking Results and System Setup

**Figure 3:**
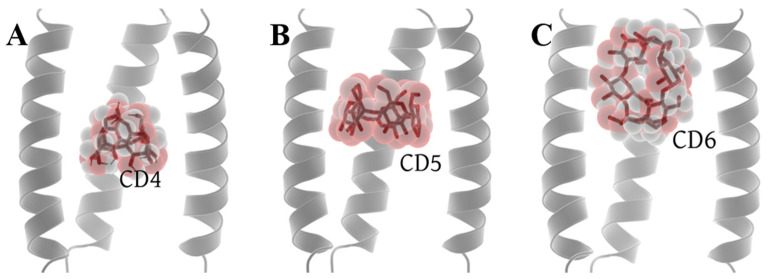
Optimal docking conformations of CDs with the ${BM2}_{TM}$ channel. (**A**) Docking conformation of CD4 with the ${BM2}_{TM}$ channel, (**B**) docking conformation of CD5 with the ${BM2}_{TM}$ channel, and (**C**) docking conformation of CD6 with the ${BM2}_{TM}$ channel.

Molecular docking was performed in the Autodock Vina platform [63] (see from the Section 3.1). Among the nine conformations generated by the docking of CD4 with 6PVT, there are two distinct positions. One is close to the entrance of the 6PVT N-terminal, and the other is above the H19 tetramer. We chose the conformation with the lowest energy from the latter position for further investigation. For CD5 docking with 6PVT, all nine conformations are predominantly located at the same position. We selected the conformation with the lowest energy for our study. In the case of CD6 docking with 6PVT, there are three different distribution positions among the nine conformations. The first resembles the binding mode of CD5 within 6PVT, the second is located outside the transmembrane domain, above the N-terminal of the 6PVT model, and the third is a vertically distributed conformation at the N-terminal of the 6PVT model’s channel, which is more prevalent. We chose the third binding mode for further investigation. One reason for this choice is that if CD6 were to bind like CD5, it would inevitably have a significant impact on the stability of the 6PVT model which tetramer diameter slightly larger than the CD6 size [15,57]. Another reason is that, in this binding mode, CD6’s larger inner diameter is not sufficient to block the passage of water molecules. Finally, we will use these three models (shown in Figure 3) for constructing our initial study models, with their respective docking affinities being −6.7 kcal/mol, −8.1 kcal/mol, and −4.4 kcal/mol (see Figure S3).

They, along with the empty proteins 6PVR and 6PVT, were inserted into a mimic membrane using CHARMM-GUI [64] (refer to Section 3.1), resulting in five ensemble models. These models include the empty ${BM2}_{TM}$ channel models 6PVT and 6PVR, as well as three complex models (6PVT_CD4, 6PVT_CD5, and 6PVT_CD6). This setup facilitates the investigation of water molecule distribution within the BM2 channel both before and after binding with cyclodextrin molecules. Additionally, it allows the examination of changes in hydrogen bonding interactions involving the serine triplet, H19, and water molecules. The ultimate goal is to elucidate the mechanism through which CDs influence the functionality of the BM2 channel.

### 2.3. Molecular Dynamics Simulation and the Stability Assessment of the Results

To investigate the impact of CDs on factors closely related to proton transfer within the BM2 channel, we conducted 200 ns all-atom molecular dynamics simulations for the five models constructed above three times, and each system has resemblance results.

**Figure 4:**
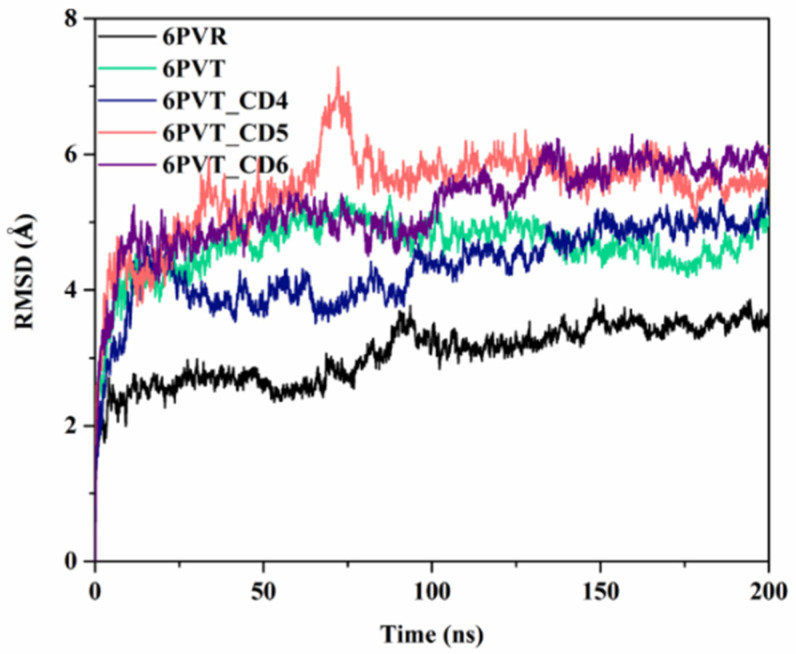
RMSD values of protein backbone atoms relative to the initial structure in various models in molecular dynamics simulations, including the 6PVR and 6PVT archetypal protein models, as well as complex models with CDs (6PVT_CD4, 6PVT_CD5 and 6PVT_CD6).

To gauge the stability of all models during the molecular dynamics simulations, we calculated the root mean square deviation (RMSD) of the C_α_ atoms of the protein backbone relative to the initial conformation for each model across the entire 200 ns simulation period (the other two parallel molecular dynamics simulations’ RMSD were appended in the Supplementary Materials Figure S5). As depicted in Figure 4, the RMSD values for all models demonstrated minimal fluctuations beyond the initial 100 ns of simulation, affirming the attainment of a stable state for each model. Consequently, all ensuing discussions and analyses in this study are rooted in the dynamic simulation trajectories of the concluding 100 ns.

### 2.4. The Binding Modes between CDs and ${BM2}_{TM}$

**Figure 5:**
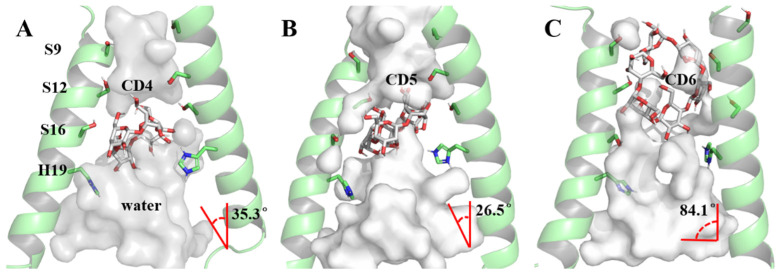
Binding modes of ${BM2}_{TM}$ channel protein with CDs and pore-lining residues. The ${BM2}_{TM}$ protein backbone is shown as a transparent green cartoon, while CDs and residues are displayed in different colored sticks. The red angle represents the angle between the multi-rotational axis of the CDs’ and the ${BM2}_{TM}$ channel’s *z*-axis. For clarity, all nonpolar hydrogens of both CDs and residues are hidden. (**A**) Binding mode of the ${BM2}_{TM}$ channel with CD4, (**B**) binding mode of the ${BM2}_{TM}$ channel with CD5, (**C**) binding mode of the ${BM2}_{TM}$ channel with CD6.

The foundation of our exploration into the impact of CD4, CD5, and CD6 on the ${BM2}_{TM}$ proton channel lies in understanding their binding modes. A clustering analysis method based on the DBScan algorithm [65,66] (see Section 3.5) was employed to discern the most prevalent conformation during the dynamic process, serving as the representative structure (Figure 5). In the case of DBScan, two hyperparameters will be defined: epsilon (ε, a distance measure that will be used to locate the points/to check the density in the neighborhood of any point) and minPoints (n, the minimum number of points (a threshold) clustered together for a region to be considered dense) to arrive at the clusters. In this work, ε is the RMSD between the structures of each trajectory, decided by the K-dist curve (see Figure S4). The ε equal to 1.0 Å was used in each system. The distinct sizes of cyclodextrin molecules led to two binding modes in the representative conformations of the 6PVT_CD4, 6PVT_CD5, and 6PVT_CD6 complex models (Figure 5). CD4 and CD5 adopted a tilted binding mode within the middle region of the ${BM2}_{TM}$. The angles between their multiple rotation axes (C_4_ and C_5_) and the ${BM2}_{TM}$ channel *z*-axis were measured at 35.3° and 26.5°, respectively (Figure 5A,B). Conversely, the larger CD6 molecule exhibited a distinct binding mode at the N-terminus of the ${BM2}_{TM}$, with its rotation axis C_6_ nearly perpendicular to the ${BM2}_{TM}$ channel *z*-axis, forming an angle of 84.1° (Figure 5C).

**Figure 6:**
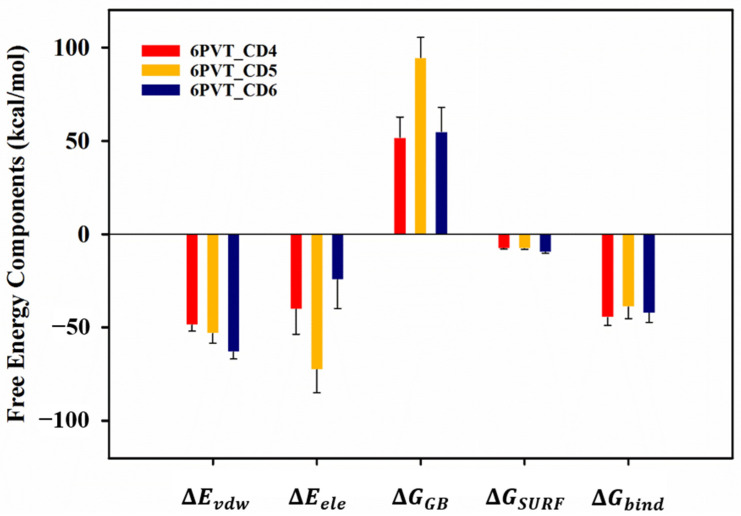
The Gibbs free energies ($\Delta G_{bind}$) and their components of CDs binding to the ${BM2}_{TM}$ proton channel. ${\Delta E}_{vdw}$, contributions by van der Waals interactions; ${\Delta E}_{ele}$, electrostatic energy; ${\Delta G}_{GB}$, polar solvation energy; ${\Delta G}_{SURF}$, nonpolar solvation energy.

To gain deeper insights into the interactions between CDs (CD4, CD5, and CD6) and the ${BM2}_{TM}$ protein, we employed the MM-GBSA method [67,68] to calculate the binding free energies. The results, outlined in Table S1, revealed binding free energies of −44.71, −38.99, and −42.24 kcal/mol for CD4, CD5, and CD6 with the ${BM2}_{TM}$ protein, respectively (refer to Table S2). These values were predominantly contributed by electrostatic and van der Waals interactions between corresponding residues in each model and the CDs (Figure 6).

To pinpoint the key residues governing the binding of the ${BM2}_{TM}$ channel with cyclodextrin molecules CD4, CD5, and CD6, we decomposed the binding free energy to obtain the residue decomposition energy for each system. Residues with a decomposition energy less than −1 kcal/mol were deemed crucial for the binding of CDs with the ${BM2}_{TM}$ channel. These residues, sorted based on their contributions to the binding energy, are listed in Tables S3–S5 in SI.

In the 6PVT_CD4 model, polar residues C_S12 (this label represents the residue S12 of the C-chain helix), D_S12, A_S16, C_S16, D_S16, A_H19, C_H19, and D_H19 as well as non-polar residues A_L15, C_L15, and D_L15 (Table S3 in SI) played pivotal roles in the binding of CD4 to the ${BM2}_{TM}$ channel. Polar residues D_S12, C_S16, and C_H19 as well as non-polar residues B_L15, C_L15, D_L15, and A_F20 (Table S4 in SI) were crucial for the binding of CD5 to the ${BM2}_{TM}$ channel. Polar residues A_S9, B_S9, A_S12, B_S12, and C_S12 as well as non-polar residues A_L8, C_L8, D_L8, B_F13, and D_L15 (refer to Table S5 in SI) played important roles in the binding of CD6 to the ${BM2}_{TM}$ channel.

The preceding analysis underscores the significance of specific residues within the ${BM2}_{TM}$ domain in mediating the binding interactions with CD4, CD5, and CD6. Notably, polar residues S12, S16, and H19, along with non-polar residue L15, positioned centrally within the transmembrane domain, play a pivotal role in facilitating the binding of CD4 and CD5 to the ${BM2}_{TM}$ protein. Conversely, for the binding of CD6 to the ${BM2}_{TM}$ domain, crucial residues include polar residues S9 and S12, along with non-polar residues L8 and L15, located at the N-terminus of the ${BM2}_{TM}$ channel. These findings shed light on the molecular determinants that underlie the specificity and stability of the interactions between CDs and the ${BM2}_{TM}$, providing valuable insights for understanding the functional implications of these interactions in the context of proton transfer within the BM2 channel.

### 2.5. CD-Containing Systems Have Discrete Water Neworks along the ${BM2}_{TM}$ Channel

In a recent study, Hong et al. utilized a combination of ss-NMR techniques and molecular dynamics simulations to delve into the orientation and motion of water molecules within the ${BM2}_{TM}$ channel [34]. Their investigation unveiled a fascinating insight into the dynamics of water molecules, suggesting that these molecules undergo rapid reorientation, breaking and forming hydrogen bonds with adjacent water molecules. This dynamic process, as explained by the Grotthuss hopping mechanism [22], facilitates proton exchange within the BM2 channel [34]. Significantly, the proposed proton transfer model bears resemblance to that observed in the proton channel of gramicidin A [69,70,71]. This correlation underscores the notion that the existence of a continuous water networks within the channel is an essential prerequisite for the efficient transfer of protons [16,26,72,73,74]. Consequently, any disruption to the continuous water network within the ${BM2}_{TM}$ channel is anticipated to impede the proton conduction.

**Figure 7:**
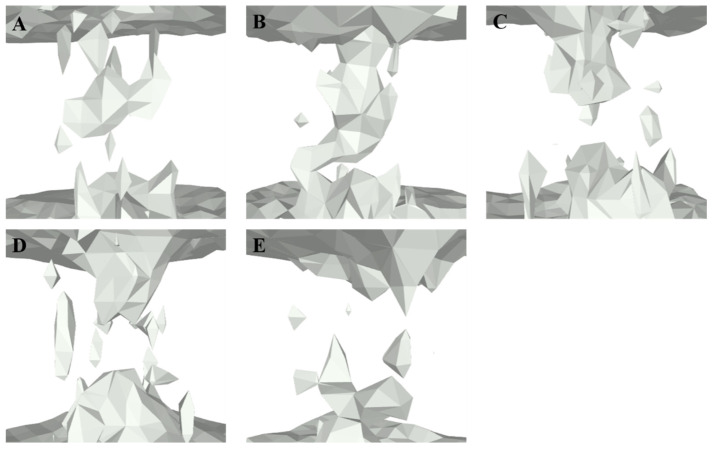
Three-dimensional distribution profiles of water molecules within the ${BM2}_{TM}$ proton channel. The top of each image corresponds to the N-terminus of the ${BM2}_{TM}$ channel, while the bottom represents the C-terminus of the ${BM2}_{TM}$ channel. The gray regions in each cartoon image represent water molecules. (**A**) 6PVR model; (**B**) 6PVT model; (**C**) 6PVT_CD4 model; (**D**) 6PVT_CD5 model; (**E**) 6PVT_CD6 model.

**Figure 8:**
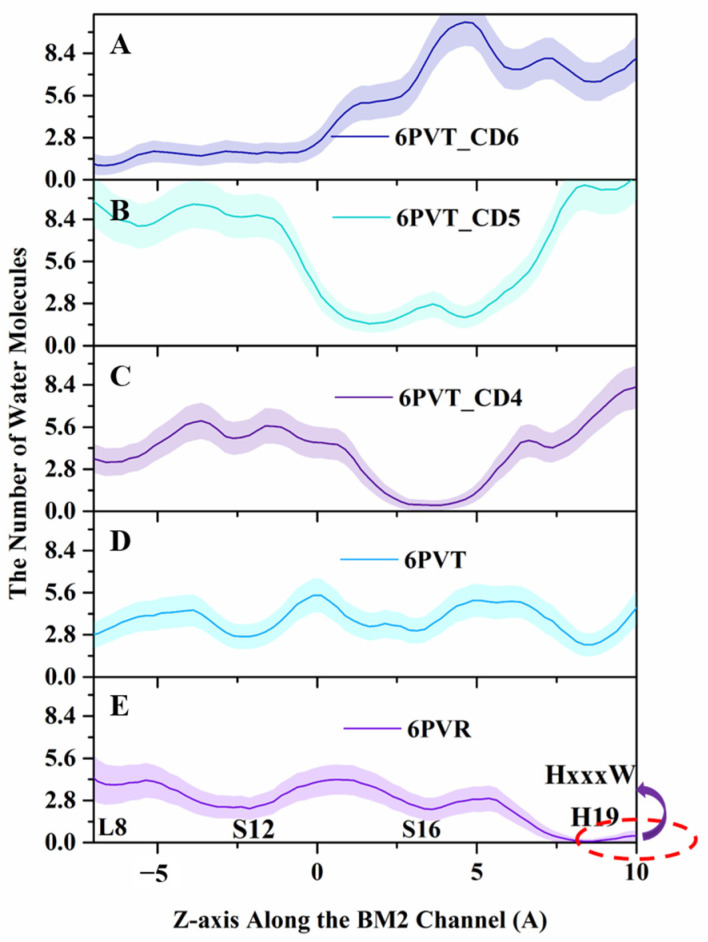
Water molecules distribution along the ${BM2}_{TM}$ channel’s *z*-axis. Lines and shadows represent the mean and standard deviation, respectively. The red dash circle represents the HxxxW motif domain of the ${BM2}_{TM}$ channel. (**A**) 6PVT_CD6 model, (**B**) 6PVT_CD5 model, (**C**) 6PVT_CD4 model, (**D**) 6PVT model, and (**E**) 6PVR model.

To clarify the influence of CDs on the distribution of water molecules in the ${BM2}_{TM}$ channel, we first calculated the three-dimensional contour of water molecule distribution in each model and the quantity of water molecules distributed along the *z*-axis of the ${BM2}_{TM}$ channel by MDAnalysis [75] and the cpptraj [76] module of AmberTools16 [77], respectively (see Figure 7 and Figure 8).

From Figure 8, it can be observed that in the archetypal ${BM2}_{TM}$ channel model 6PVR, the quantity of water molecules near the HxxxW motif domain is close to zero (shown as Figure 8E). This phenomenon may be attributed to the π-π interaction between residues H19 and W23, hindering the diffusion of water molecules in that region [34,78]. On the contrary, in the activated archetypal ${BM2}_{TM}$ channel model 6PVT, the water molecules are continuously distributed. In the 6PVT_CD4 model, the number of water molecules near the CD4 binding site is close to zero, primarily located between the S16 tetramer (shown as Figure 8C). Similarly, in the 6PVT_CD5 model, between the S12 and S16 tetramers, corresponding to the CD5 binding site, there is a point with the lowest quantity of water molecules (shown as Figure 8D). The 6PVT_CD6 model has the lowest number of water molecules near the L8 tetramer, maintaining a consistently lower level of water molecules between the L8 and S12 tetramers. The data above indicate that, compared to the archetypal model 6PVT, all complex models exhibit a varying degree of reduction in the quantity of water molecules at CDs binding sites, separating the hydrogen-bonded networks of water molecules into two completely isolated parts (see Figure 7).

It is worth noting that at the CD4 binding site, the continuous water networks are almost completely blocked, and the effect of CD4 blocking the continuous hydrogen-bonding network is similar to that of the archetypal model 6PVR.

Lately, numerous studies have consistently indicated that the enlargement of the BM2 channel pore and thorough hydration are favorable factors for proton transfer [15,16,34], and the presence of a continuous water networks in the channel is an indispensable condition for proton transfer [20,34]. Therefore, we can infer that the complex models binding with CDs cause a narrowing of the water channel, making the diffusion of water molecules in the channel difficult. This may be the fundamental reason for the rupture of continuous water networks in the channel of complex models.

### 2.6. CD-Containing Systems Have a Narrowed Water Channel and Are Difficult for Water Molecules to Pass Through

To confirm this inference, we conducted analyses of two aspects: the variation in pore radius along the channel in each model and the change in energy barrier for water molecules diffusion in these models’ channels.

**Figure 9:**
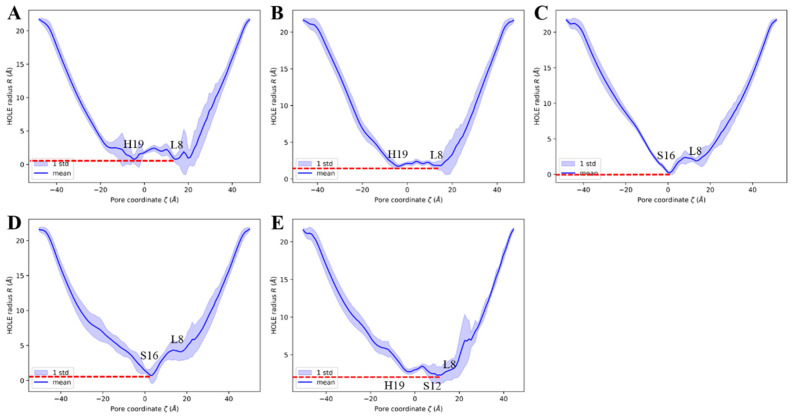
The channel pore radius along the *z*-axis, the line and the shade represent the mean value and the standard deviations, respectively. The red dashed line indicates the lowest mean value. (**A**) The 6PVR system. (**B**) The 6PVT system. (**C**) The 6PVT_CD4 system. (**D**) The 6PVT_CD5 system. (**E**) The 6PVT_CD6 system.

To elucidate the impact of CDs bound within the ${BM2}_{TM}$ channel on the pore size of complex models, we calculated the variations in pore radius along the channel for all models by the HOLE2 [79] in MDAnalysis [75], as depicted in Figure 9. The calculations encompass the entire *z*-axis range of the models, with the interval approximately ranging from −20 to 20 Å, representing the transmembrane region of the channel.

The empty protein model 6PVR exhibits a bottleneck with a radius of approximately 0.8 Å near the L8 and H19 tetramers (shown as Figure 9A). This finding aligns with Hong et al.’s NMR experiments combined with molecular dynamics simulations, suggesting that L8 and H19 serve as bottleneck regions within the BM2 channel [34]. Similarly, the empty protein model 6PVT shows pore diameters of about 1.9 Å near the L8 and H19 tetramers, with L8 and H19 remaining the two narrowest positions in the 6PVT model.

In the complex model with CD4, the narrowest point in the channel is near the S16 tetramer, with a channel clearance radius approaching zero (see Figure 9C), potentially impeding the distribution of water molecules in this region. The complex model with CD5 exhibits a minimum channel clearance at a position near the S16 tetramer, similar to the CD4 complex model, with a clearance radius of approximately 0.8 Å (see Figure 9D). The complex model with CD6 exhibits a minimum channel clearance radius of approximately 1.9 Å near the S9 tetramer (refer to Figure 9E), resulting from a gap formed between CD6 and the ${BM2}_{TM}$ channel due to the binding mode of CD6 (as illustrated in Figure 5C). This gap may permit the passage of a few water molecules through the ${BM2}_{TM}$ channel (depicted in Figure 8A).

For all models with CDs, the minimum pore positions are roughly located at their respective binding sites, consistent with the points corresponding to the lowest quantity of water molecules (see Figure 8). These data indicate that, compared to the empty protein model 6PVT, the models with CD4 and CD5 have smaller pores at their binding sites. Particularly noteworthy is the CD4 complex model, which almost entirely obstructs the BM2 channel near the CD4 binding site.

**Figure 10:**
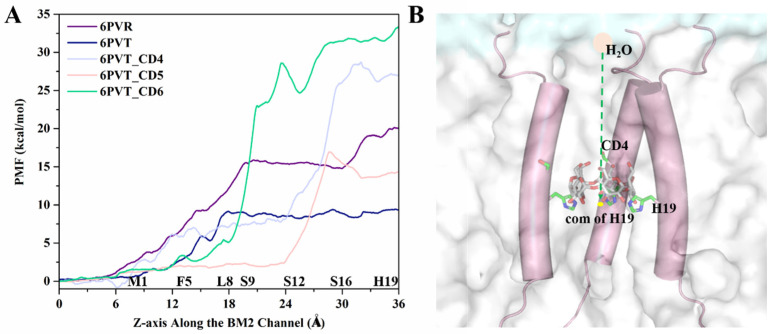
ASMD simulation of the water molecule diffusion process. (**A**) PMF variation curve depicting the water molecule diffusion process within the ${BM2}_{TM}$ channel. (**B**) Schematic representation, using the ASMD method, of the movement of water molecules from the N-terminus of the ${BM2}_{TM}$ channel to the center of mass (COM) of the H19 tetramer. Water molecules are depicted as pink circles, the ${BM2}_{TM}$ channel skeleton as cylinders, lipid molecules as transparent white surfaces, yellow dots represent the COM of the H19 tetramer, and CD5 and H19 are depicted as sticks.

In addition, the Adaptive Steered Molecular Dynamics (ASMD) method was employed to simulate the diffusion process of water molecules at the N-terminus of the ${BM2}_{TM}$ channel. The Jarzynski equation [80] was utilized to calculate the changes in the diffusion barriers during the process, and Potential of Mean Force (PMF) curves were generated (refer to Figure 10A). In the simulation, a water molecule was pulled from a position 10 Å away from the N-terminus of the ${BM2}_{TM}$ proton channel along the central *z*-axis of the channel to the position of the H19 tetramer’s center of mass (COM). As depicted in Figure 10A, the range from 16 Å to 36 Å corresponds to the transmembrane structural domain of the BM2 channel. Water molecules encounter greater restrictions in diffusion in this region compared to the region outside the transmembrane structural domain of the BM2 channel (0 to 16 Å in Figure 10A). This result aligns with previous experimental findings [26,81,82,83].

From Figure 10, the primary distinction in the PMF curves of these five models is observed behind the L8 tetramer. Let us first analyze the differences in this region between the empty protein models 6PVR and 6PVT. In this region, the 6PVR model’s PMF values exhibit two changing stages. The first stage, within the L8 to S16 residue range, maintains a barrier at approximately 15 kcal/mol, while the second stage, in the S16 to H19 residue range, sees the barrier rise to about 20 kcal/mol. This might be associated with the π-π interaction formed between residues H19 and W23, hindering the diffusion of water molecules in this region [34,78]. In contrast, the 6PVT model shows only one stage, with PMF values maintaining a level of about 8 kcal/mol. The difference in the diffusion barriers for water molecules in the BM2 channel under different states is also reflected in the BM2 channel’s ability to transfer protons according to the Grotthuss mechanism during its active state [34].

Now, let us delve into the PMF variations in all complex models with bound CDs. From Figure 10A, it can be observed that, after the L8 tetramer, models with bound CDs all experience a rapid increase in PMF values. Except for the model with CD5, the PMF values of the other two complex models in this region are significantly higher than those of the two empty protein models. The model with CD4 exhibits the highest barrier, reaching up to 27 kcal/mol, and this barrier is located around the binding cavity of CD4, between residues S12 and H19 tetramers. This is closely related to the smaller channel clearance in this region (see Figure 9C). In the model with CD5, there is a small barrier of about 2 kcal/mol before the S12 tetramer, corresponding to the larger channel clearance measured in this range (see Figure 9D). The barrier rapidly rises to 15 kcal/mol between S16 tetramers, corresponding to the position where CD5 binds to BM2. Except for this part, the barriers in other regions are lower than those in the corresponding regions of the 6PVR model. This observation aligns with the binding mode of CD5 and its relatively large size, tilting within the ${BM2}_{TM}$ channel, enlarging the radius of the ${BM2}_{TM}$ channel (Figure 9D), thereby increasing the diffusion space for water molecules. This makes water molecules more likely to diffuse through areas of the ${BM2}_{TM}$ channel, except for the binding position of CD5, resulting in the observed characteristics of water molecule diffusion PMF. These results align with previous studies indicating that the size of the BM2 channel radius directly influences the flux of water molecules in the channel [15,34]. After the L8 tetramer, when water molecules pass through the ${BM2}_{TM}$ channel bound to CD6, the barrier remains high. In contrast, there is a relatively low barrier at the position of the S12 tetramer, determined by the binding mode of CD6 in the ${BM2}_{TM}$ channel and its larger hydrophobic cavity (see Figure 5C). As water molecules traverse the hydrophobic cavity of CD6 in the ${BM2}_{TM}$ channel, the PMF value rapidly increases to approximately 35 kcal/mol.

It is noteworthy that the measured channel clearance radius indicates that the channel of model with CD6 exhibits a gap, potentially allowing a small amount of water molecules to pass through (Figure 8 and Figure 9). However, the PMF of water molecule diffusion measured at the binding position of CD6 remains high (see Figure 10A). This is because, as mentioned before, the steered process was conducted along the central *z*-axis of the channel of all models. The path of water molecules traverses the space occupied by CD6, leading to the observed high PMF values, as illustrated in Figure 10A.

The analysis results presented above indicate that the binding of CDs results in a reduction of the pore radius of the ${BM2}_{TM}$ channel, creating a more confined space that hinders water molecule diffusion. This restriction poses a challenge for water molecules to traverse the ${BM2}_{TM}$ channel efficiently, leading to the disruption of continuous water networks within the water channel and restricting the conduction of protons [34]. Of particular interest is the binding mode of CD5 in the 6PVT_CD5 model, where CD5 exhibits a tilted binding manner to the ${BM2}_{TM}$ channel and also features a relatively larger hydrophobic interior, facilitating the passage of water molecules through the channel (refer to Figure 10A). CD6 introduces a small gap between itself and the ${BM2}_{TM}$ channel, potentially permitting a limited number of water molecules to traverse the channel (refer to Figure 5C and Figure 8A). On the contrary, CD4 completely obstructs the ${BM2}_{TM}$ channel, impeding the flow of water molecules. Considering these observations, CD4 emerges as a more promising candidate inhibitor for the BM2 proton channel. To bolster this speculation, the subsequent section will delve into whether the disturbance of continuous water networks, resulting from the binding of CDs to the ${BM2}_{TM}$ channel, influences the interactions between the serine triads and H19 with solvent molecules. This exploration aims to shed light on the potential impact of disrupted water networks on the effective conduction of protons within the BM2 channel.

### 2.7. Decreased Pore Hydration and Disrupted Hydrogen Bond

The interaction between water molecules and the residues of serine triplet and H19 closely involved in proton transfer within the BM2 channel is pivotal for effective proton conduction [19,21,34,78,84,85]. The serine triad, positioned on the ${BM2}_{TM}$ channel’s inner surface, coordinates water molecules [19,20], forming continuous hydrogen-bonding networks that facilitate proton conduction [15,19,21,78], while titratable histidine H19 is responsible for shuttling protons through the formation of hydrogen bonds with water molecules [16,21,22,34]. In other cases, such as bacteriorhodopsin, similar hydrogen-bonding networks can be established through titratable amino acid side chains to facilitate proton conduction [86]. In the AM2 channel, protons are conveyed through a mixed hydrogen-bonding chain between water molecules and histidine residues [84,85]. To gain deeper insights into how CDs binding to the ${BM2}_{TM}$ channel affects the interaction between serine triplet and H19 with water molecules involved in proton conduction, this section analyzes the Solvent Accessible Surface Area (SASA) of the serine triad and H19 residues, along with their hydrogen bond occupancy with water molecules. SASA represents the size of the area a residue contacts with water molecules and is closely associated with the hydration of residues; a larger SASA typically indicates more hydrophilic residues on the protein surface, providing more opportunities to form hydration interactions with water molecules [87].

**Figure 11:**
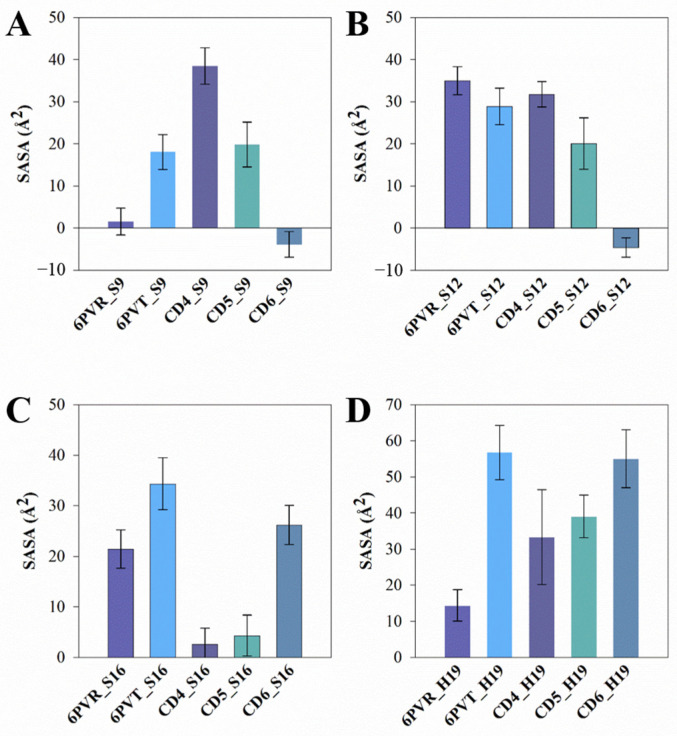
The average SASA of the polar residue tetrad on the surface of the ${BM2}_{TM}$ channel. The standard deviation is presented as error bars in the figures. (**A**) SASA of residue S9 in each system; (**B**) SASA of residue S12 in each system; (**C**) SASA of residue S16 in each system; (**D**) SASA of residue H19 in each system.

The analysis results (Figure 11 and Figure S1) demonstrate a reduction in SASA for corresponding residues in models with bound CDs, indicating a decreased probability of these residues coming into contact with solvent molecules, consequently affecting their hydrogen bond occupancy with water molecules. The following provides a detailed discussion of these changes and their underlying reasons.

**Figure 12:**
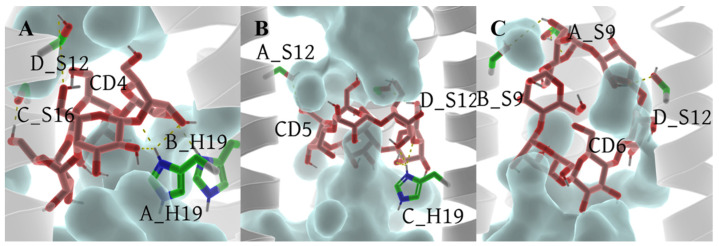
Hydrogen bonds between CDs and the ${BM2}_{TM}$. Hydrogen bonds formed between CDs and residues are shown as yellow dashed lines. The tetramer backbone is displayed as a gray transparent helix. Water molecules are depicted as a transparent cyan surface. CDs and residue serines and H19 are displayed as sticks. For clarity, all nonpolar hydrogens of both CDs and residues are hidden. (**A**) 6PVT_CD4 model, (**B**) 6PVT_CD5 model, and (**C**) 6PVT_CD6 model.

In comparison to the archetypal model 6PVT, the SASA of residue S16 significantly decreases in the 6PVT_CD5 and 6PVT_CD4 model (Figure 11C and the red-boxed S16 tetramer SASA in Figure S1B–D). Simultaneously, in these two complex models, the hydrogen bond occupancy between residue A_S16 and solvent molecules noticeably decreases (see Table S9). Furthermore, in these two complex models, the SASA of residue H19 decreases by approximately 30% (Figure 11D and the green-boxed H19 tetramer SASA in Figure S1B–D). In the 6PVT_CD4 model, the occupancy of hydrogen bonds formed by A_H19 and B_H19 with solvent molecules significantly decreases compared to the 6PVT model (Figure 11). This is closely related to the binding mode of CD4 to ${BM2}_{TM}$ channels (Figure 5A) and the hydrogen bonds formed between CD4 and residues on the ${BM2}_{TM}$ channel (Figure 12A and Table S6). CD4 forms strong electrostatic interactions with A_H19 in the BM2 channel (Table S3), and CD4 forms hydrogen bonds with both A_H19 and B_H19 (see Table S6 and Figure S2A). These interactions anchor CD4 in the middle of the ${BM2}_{TM}$ channel, occupying the distribution space of solvent molecules in this region. Consequently, this affects the probability of contact between S16 and H19 tetramers with solvent molecules, ultimately reducing their hydrogen bond occupancy with solvent molecules. In this scenario, it becomes challenging for the serine residues to coordinate water molecules [19,20], sustaining continuous hydrogen-bonding networks that facilitate proton conduction [15,19,21,78]. In the 6PVT_CD5 model, the occupancy of hydrogen bonds formed by C_H19 with solvent molecules noticeably decreases (Table S9). This is related to the binding mode of CD5 to ${BM2}_{TM}$ channels (Figure 5B) and the hydrogen bonds formed between CD5 and residue C_H19 (Figure 12B and Table S7). CD5 exhibits strong electrostatic interactions with C_H19 residues in the BM2 channel (Table S4). Additionally, CD5 forms hydrogen bonds with residue C_H19 (see Table S7). This, to some extent, explains the significant decrease in the hydrogen bond occupancy between residue C_H19 and solvent molecules in the 6PVT_CD5 model.

The binding mode of CD6 to ${BM2}_{TM}$ channels differs from CD4 and CD5, as it binds to the N-terminus of the ${BM2}_{TM}$ domain, mainly forming favorable interactions with the polar residues S9 and S12 within the ${BM2}_{TM}$ channel. The SASA of both S9 and S12 tetramers significantly decreases compared to the empty protein model 6PVT (see Figure 11A,B and the red-boxed SASA of S9 and S12 tetramers in Figure S1B,E). Simultaneously, the hydrogen bond occupancy between S9 tetramers and solvent molecules decreases significantly or is completely eliminated compared to the archetypal model 6PVT (see Table S9). Furthermore, compared to the archetypal model 6PVT, in the CD6 complex model, the occupancy of hydrogen bonds formed by residues C_S12 and D_S12 with solvent molecules also decreases significantly. These phenomena are closely associated with the binding mode of CD6 to ${BM2}_{TM}$ channels (see Figure 5C) and the hydrogen bonds formed between CD6 and corresponding residues within the ${BM2}_{TM}$ channel (Figure 12C and Table S8). Residues A_S12 and B_S9 in the ${BM2}_{TM}$ channel form van der Waals interactions with CD6, and A_S9 forms electrostatic interactions with CD6 (Table S5). Additionally, CD6 forms hydrogen bonds with residues A_S9, B_S9, and D_S12 (Figure 12C and Table S8). These interactions anchor CD6 at the N-terminus of the ${BM2}_{TM}$ channel, occupying the distribution space of solvent molecules in this region. Consequently, this affects the probability of contact between S9 and S12 tetramers with solvent molecules, ultimately reducing their hydrogen bond occupancy with solvent molecules. In this scenario, serine residues struggle to form stable hydrogen bonds with water molecules, impeding the maintenance of continuous water networks favorable for proton transfer within the BM2 channel [20].

It is noteworthy that in all complex models with bound CDs, there is a characteristic feature of hydrogen bonds formed by the H19 tetramer with solvent molecules. These hydrogen bonds, regardless of their varying occupancy, belong to hydrogen bonds formed between water molecules at the C-terminus of the ${BM2}_{TM}$ channel and the H19 tetramer. This is because, after CDs binding, water molecules in the ${BM2}_{TM}$ channel are separated by CDs into two isolated parts (Figure 7). Water molecules at the N- and C-terminus of the ${BM2}_{TM}$ channel struggle to form continuously connected hydrogen-bonding network. Even if the hydrogen bond occupancy between the H19 tetramer and solvent molecules is high, it cannot achieve the transfer of protons from the water molecules at the N-terminus of the ${BM2}_{TM}$ channel to the water molecules at the C-terminus [19,20]. This ultimately inhibits the effective transfer of protons within the BM2 channel.

## 3. Materials and Methods

### 3.1. Molecular Docking and System Construction

Molecular docking, a common method for predicting ligand-receptor binding modes [88], was employed to predict the binding conformations of CD4, CD5, and CD6 with the ${BM2}_{TM}$ channel protein. The docking conformations were obtained using the Autodock Vina platform [63].

The transmembrane domain of the BM2 channel protein is embedded in the phospholipid bilayer [15,89], making it challenging for cyclodextrin molecules to interact with the periphery of the BM2 channel protein. Additionally, the HxxxW motif in the transmembrane domain of the BM2 channel protein is relatively narrow [15,34], making it difficult for cyclodextrin molecules to pass through this region of the BM2 channel. Considering these circumstances, we only need to examine the binding of cyclodextrin molecules to the N-terminal of the transmembrane domain of the BM2 channel. We configured docking boxes to fully encapsulate the N-terminal portion of the BM2 transmembrane domain, with sizes specified in Table S10. Given that proton transfer in the BM2 channel occurs under acidic conditions [14,15], the activated state structure of the ${BM2}_{TM}$ channel (6PVT) was docked with structurally relaxed CDs (CD4, CD5, and CD6). Each cyclodextrin molecule in the 6PVT model generates 9 docking conformations. The optimal docking conformations, selected based on affinity scores and spacial poses (Figure S3), were used for subsequent analysis (shown as Figure 3).

Utilizing CHARMM-GUI [64], the optimal docking conformations as well as two empty proteins 6PVR (closed state) and 6PVT (open state) mentioned above [15] were embedded into a negatively charged membrane environment composed of 165 lipid molecules, shown in Table S1 in supporting information (SI) in a molar ratio of 60% POPC, 20% POPG, and 20% cholesterol [21], mirroring eukaryotic membranes [19,90]. Additionally, two layers of water, each 15 Å thick, were added on both sides of the lipid bilayer, and 40 Na^+^ and 19 Cl^−^ ions were included to maintain physiological salt concentration and system neutrality. This process yielded five ensemble models, encompassing empty ${BM2}_{TM}$ channel models 6PVT and 6PVR, as well as three complex models (6PVT_CD4, 6PVT_CD5, and 6PVT_CD6). The number of water molecules and the size of each models were appended in Supplementary Tables S11 and S12.

### 3.2. Molecular Dynamics Simulations

In model construction, the ${BM2}_{TM}$ channel protein used the ff14SB force field [91], lipid molecules adopted the lipid14 force field [92], and water molecules followed the TIP3P model [93]. The GAFF2 force field was used for CDs in Amber16 [94] using the antechamber module [95]. Simulation input files were generated using the *tleap* module of Amber16 [77,94].

To study the impact of CDs on the activated state of the BM2 proton channel, molecular dynamics simulations were performed on the five models constructed previously (empty protein models for 6PVR and 6PVT, and complex models for 6PVT_CD4, 6PVT_CD5, and 6PVT_CD6). The simulations adopted periodic boundary conditions and utilized the PME [96] method for long-range electrostatic interactions, with a cutoff radius set at 10 Å. The SHAKE algorithm [97,98] was employed to constrain all hydrogen atoms. The integration time step was set to 2 fs. Before initiating molecular dynamics simulations, each model underwent two rounds of energy minimization, including 10,000 steps of steepest descent and 10,000 steps of conjugate gradient minimization. The first minimization applied a constraint force of 100 kcal/mol/Å^2^ solely to the backbone carbon atoms of the ${BM2}_{TM}$ channel protein. In the second minimization, all atoms were unconstrained. Subsequently, each system was gradually heated to 310 K and underwent a 5 ns dynamics equilibrium process. Finally, in an NPT ensemble (thermostat is using Langevin dynamics with the collision frequency in 1 ps^−1^, barostat is using Monte Carlo barostat to regulate the pressure of ensembles) and periodic boundary conditions, each model underwent a 200 ns molecular dynamics simulation, repeated three times. The total sampling time for the five models reached 3 μs. Trajectories were saved every 20 ps for subsequent analysis. All simulations were conducted in AMBER16 [94].

### 3.3. Binding Free Energy Calculation

Based on the molecular dynamics simulation results, the MM-GBSA method [99,100,101] was employed to calculate the binding free energies of CD4, CD5, and CD6 with the ${BM2}_{TM}$ channel, along with their corresponding residue decomposition energies. The 1000 frames used for calculations were extracted from the last 100 ns of equilibrium trajectories.

For each snapshot, the binding free energy ($\Delta G_{bind}$) of the protein and ligand was calculated the following formulas:

(1) $$\Delta G_{bind}=\Delta G_{complex}-\Delta G_{receptor}-\Delta G_{ligand},$$

$\Delta G_{complex}$, $\Delta G_{receptor}$ and $\Delta G_{ligand}$ are the free energy of the complex, receptor, and ligand, respectively. The binding free energy can also be described as listed equation below:

(2) $$\Delta G_{bind}=\Delta G_{gas}+\Delta G_{solv},$$

$\Delta G_{gas}$ and $\Delta G_{solv}$ are the solvation energy and non-solvation energy, respectively.

(3) $$\Delta G_{gas}{=\Delta E}_{bond}+{\Delta E}_{angle}+{\Delta E}_{dihed}+{\Delta E}_{vdw}+{\Delta E}_{ele},$$

(4) $$\Delta G_{solv}={\Delta G}_{GB}+{\Delta G}_{SURF},$$

The energy term $\Delta G_{gas}$ of the molecular mechanics can be divided into the ${\Delta E}_{bond}$ (bond energy), ${\Delta E}_{angle}$ (bond angle energy), ${\Delta E}_{dihed}$ (dihedral angular energy), ${\Delta E}_{ele}$ (electrostatic energy), and ${\Delta E}_{vdw}$ (van der Waals energy). In addition, both polar solvation energy (${\Delta G}_{GB}$) calculated by generalized Born (GB) model and the non-polar solvent interaction (${\Delta G}_{SURF}$) contribute to the solvation energy ($\Delta G_{solv}$).

(5) $${\Delta G}_{SURF}=\gamma SASA+b,$$

The ${\Delta G}_{SURF}$ is calculated from surface tension (γ) and solvent accessible surface area (SASA) which computed by the fast LCPO algorithm [102]. The γ and b were set as 0.0072 kcal/mol/Å^2^ and 0.92 kcal/mol, respectively.

To recognize the key residues by per-residue energy contribution, the energy decomposition analysis was also calculated by the MM-GBSA method.

(6) $$\Delta G_{bind}^{per-residue}=\Delta G_{gas}^{per-residue}+\Delta G_{solv}^{per-residue},$$

$\Delta G_{gas}^{per-residue}$ and $\Delta G_{solv}^{per-residue}$ are the same terms as depicted in MM-GBSA calculation.

### 3.4. Adaptive Steered Molecular Dynamics (ASMD) Simulations

To assess the impact of CDs on water molecule diffusion in the ${BM2}_{TM}$ channel, ASMD simulations [103,104,105] were conducted to obtain the changes in the potential of mean force (PMF) as water molecules traversed the channel. Water molecules were pulled along the *z*-axis of the ${BM2}_{TM}$ channel from a position 10 Å away from the N-terminus to the centroid of the H19 tetramer (see Figure 10B). The steering speed and protein force constant were set to 4 Å/ns and 100 kcal/mol/Å^2^, respectively. The simulation trajectory was divided into 18 segments, each repeated 10 times. Using the Jarzynski equality [106], non-equilibrium work was obtained to derive the PMF along the reaction coordinate.

(7) $$G(\xi_{t})=G(\xi_{0})-\frac{1}{\beta }\ln{\langle e^{-\beta W_{\xi_{t}{\leftarrow \xi }_{0}}}\rangle }_{0},$$

Here, $W_{\xi_{t}{\leftarrow \xi }_{0}}$ represents the work done from state $\xi_{0}$ to state $\xi_{t}$, $\beta ={{(k}_{B}T)}^{-1}$, where $k_{B}$ and T are the Boltzmann constant and temperature, respectively.

### 3.5. Analysis Methods

We obtained the representative conformations from cluster [66] by *DBScan* algorithm [65]. *DBScan* is a clustering algorithm that defines clusters as continuous regions of high density and works well if all the clusters are dense enough and well separated by low-density regions.

Algorithms start by picking a point (one record) x from dataset at random and assign it to a cluster 1. Then, it counts how many points are located within the *ε* distance from x. If this quantity is greater than or equal to minPoints (n), it then considers it a core point, and then it will pull all these *ε*-neighbors out to the same cluster 1. It will then examine each member of cluster 1 and find their respective *ε*-neighbors. If some member of cluster 1 has n or more *ε*-neighbors, it will expand cluster 1 by putting those *ε*-neighbors into the cluster. It will continue expanding cluster 1 until there are no more examples to put into it. In the latter case, it will pick another point from the dataset not belonging to any cluster and put it to cluster 2. It will continue like this until all examples either belong to some cluster or are marked as outliers.

If a small *ε* is chosen, a large part of the data will not be clustered, whereas for a too high value of *ε*, clusters will merge, and the majority of objects will be in the same cluster. Hence, the value for *ε* can then be chosen by using a K-dist graph (https://www.mygreatlearning.com/blog/dbscan-algorithm/ (accessed on 14 July 2023)).

The number of water molecules was calculated from the cpptraj [76] module action command density of Amber16 package [77] within a 0.25 Å bin along the *z*-axis of ${BM2}_{TM}$ proton channel. Water molecules are assigned based on the average position of their oxygen atom all over the trajectories.

The pore dimensions were analyzed by the HOLE2 [79] in MDAnalysis package [75]. The solvent density around ${BM2}_{TM}$ were also analyzed in MDAnalysis package [75].

## 4. Conclusions

The binding of CD4, CD5, and CD6 to the ${BM2}_{TM}$ channel significantly alters the distribution of water molecules within their respective binding regions, consequently affecting the contact area between the serine triplet and H19 with water molecules. This leads to a notable reduction in the SASA of the serine triplet and H19, accompanied by a decrease in hydrogen bond occupancy crucial for proton transfer [19,21,78,107,108]. These combined changes hinder the effective transfer of protons within the BM2 channel, suggesting that CD4, CD5, and CD6 individually contribute to diminishing the proton transfer efficiency of the BM2 protein.

CD5, featuring a relatively larger hydrophobic interior, displays a tilting binding configuration in the ${BM2}_{TM}$ channel, making it slightly easier for water molecules to pass through. CD6 binds perpendicularly to the ${BM2}_{TM}$ channel with a small gap between them, potentially allowing a limited number of water molecules to traverse the channel. In contrast, CD4 completely obstructs the ${BM2}_{TM}$ channel, impeding the diffusion of water molecules across it. Consequently, CD4 demonstrates promising potential as an inhibitor for the BM2 proton channel.

In summary, this research provides a comprehensive understanding of the microscopic-level binding modes and interaction details between CDs and the BM2 channel. These insights offer valuable theoretical guidance for future drug development efforts targeting the BM2 proton channel.

## Data Availability

Data is available in the manuscript.

## Supplementary Materials

The following supporting information can be downloaded at: https://www.mdpi.com/article/10.3390/molecules29030620/s1, Figure S1. The SASA of serine triads and the H19 tetramer on the inner surface of the ${BM2}_{TM}$ channel tetramer. The standard deviation is shown as error bars. (A) the 6PVR model, (B) the 6PVT model, (C) the 6PVT_CD4 model, (D) the 6PVT_CD5 model, and (E) the 6PVT_CD6 model; Figure S2. The hydrogen bond lifetime of (A) CD4-containing and (B) CD5-containing model; Figure S3. Docking affinity of CD4, CD5 and CD6 with the 6PVT model, respectively; Figure S4. The Kdist of each system. (A) the 6PVT_CD4 system, (B) the 6PVT_CD5 system, and the 6PVT_CD6 system; Figure S5. RMSD values of protein backbone atoms relative to the initial structure in the other two parallel molecular dynamics simulations, including the 6PVR and 6PVT archetypal protein models, as well as complex models with CDs (6PVT_CD4, 6PVT_CD5 and 6PVT_CD6); Figure S6. The per-lipids area of each frame; Figure S7. Schematic representation of conventional and HPγCD-curcumin liposomes (refer in article [49]). Table S1. The number of lipids of each model; Table S2. Binding Free Energy for the Three Complex Models: 6PVT_CD4, 6PVT_CD5, and 6PVT_CD6 (kcal/mol); Table S3. In the 6PVT_CD4 model, the residue decomposition energy of key residues (kcal/mol); Table S4. In the 6PVT_CD5 model, the residue decomposition energy of key residues (kcal/mol); Table S5. In the 6PVT_CD6 model, the residue decomposition energy of key residues (kcal/mol); Table S6. The hydrogen bond between CD4 and the residues of BM2 that occupancy more than 20%; Table S7. The hydrogen bond between CD5 and the residues of BM2 that occupancy more than 20%; Table S8. The hydrogen bond between CD6 and the residues of BM2 that occupancy more than 20%; Table S9. The occupancy of hydrogen bonds formed between residues Serine triplet and H19 with solvent molecules; Table S10. The centers and sizes of docking boxes in x, y, and z dimensions (Å); Table S11. The water molecules number of each system; Table S12. The model size of each system; Table S13. Parameters of lipid solubility and water solubility of CD4, CD5, and CD6; The partial charges of each atom of cyclodextrin molecules.
