# Supplementary material for: The Potential of Cyclodextrins as Inhibitors for the BM2 Protein: An In Silico Investigation
Source: Molecules. 2024 Jan 28;29(3):620. doi: 10.3390/molecules29030620 (PMC10856705; doi:10.3390/molecules29030620)
Supplement: Supplementary file 1 [file molecules-29-00620-s001.zip › molecules-2807408-supplementary.pdf]

Article

The Potential of Cyclodextrins as Inhibitors for the BM2 Protein: An in Silico Investigation

Aijun Liu [1,†], Hao Zhang [1,†], Qingchuan Zheng [2,*] and Song Wang [1,*]

[1] Institute of Theoretical Chemistry, College of Chemistry, Jilin University, Changchun 130023, China; liuaj20@mails.jlu.edu.cn (A.J.); stringbell@jlu.edu.cn (H.Z.); ws@jlu.edu.cn (S.W.)
[2] School of Pharmaceutical Sciences, Jilin University, Changchun, 130021, China; zhengqc@jlu.edu.cn (Q.C.)
* Correspondence: zhengqc@jlu.edu.cn (Q.C.) and ws@jlu.edu.cn (S.W.)
† These authors contributed equally to this work.

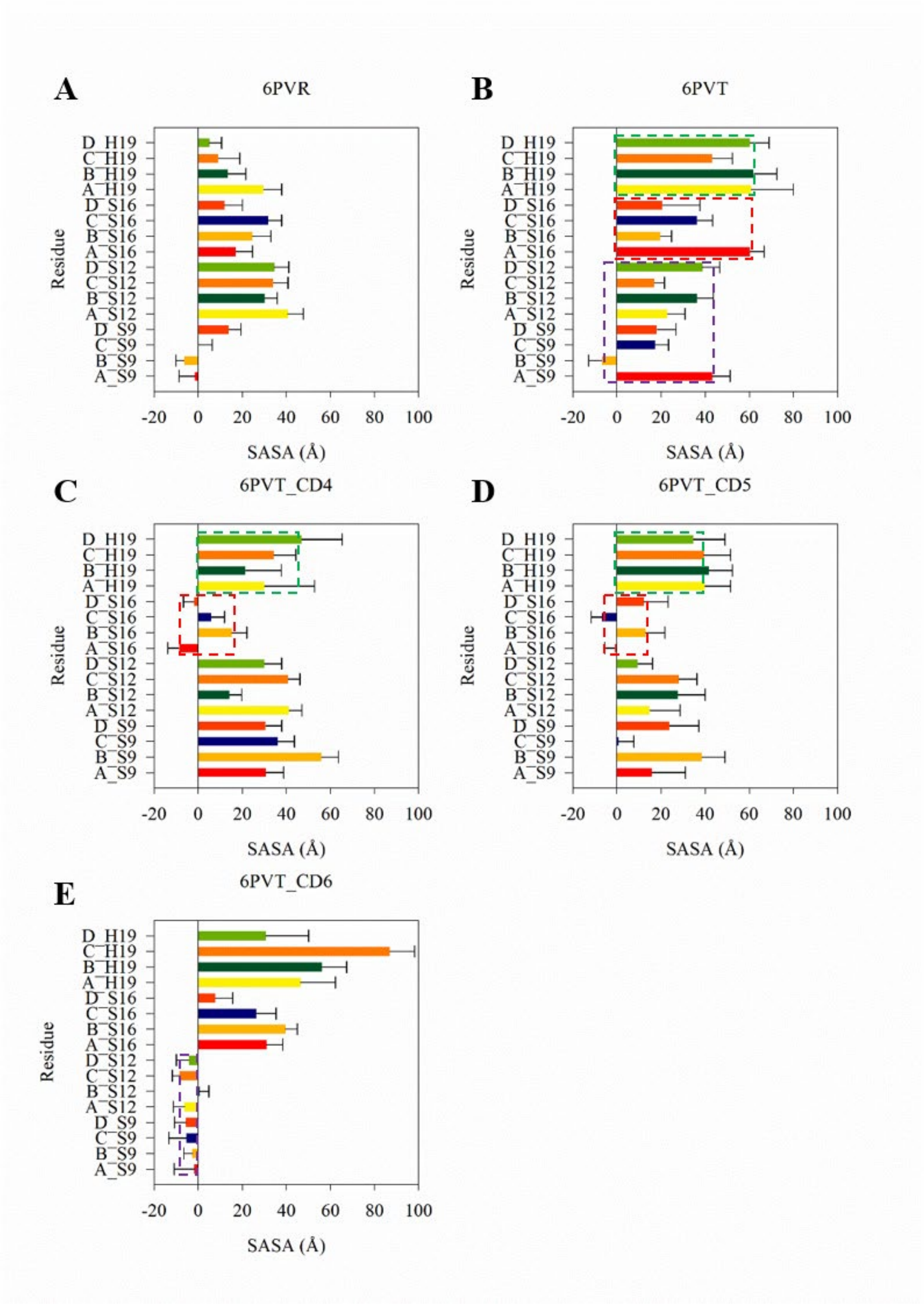

Figure S1. The SASA of serine triads and the H19 tetramer on the inner surface of the $BM2_{TM}$ channel tetramer. The standard deviation is shown as error bars. (A) the 6PVR model, (B) the 6PVT model, (C) the 6PVT_CD4 model, (D) the 6PVT_CD5 model, and (E) the 6PVT_CD6 model.

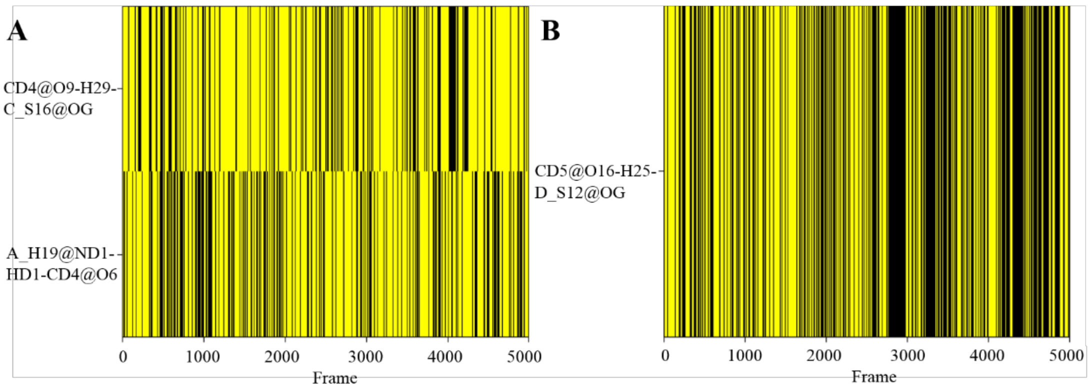

Figure S2. The hydrogen bond life time of (A) CD4-containing and (B) CD5-containing model.

mode	affinity (kcal/mol)	dist from best mode rmsd l.b.	rmsd u.b.
1	-6.8	0.000	0.000
2	-6.7	1.609	5.453
3	-6.7	1.736	6.371
4	-6.7	8.138	12.178
5	-6.7	8.077	12.062
6	-6.7	8.046	11.985
7	-6.7	1.512	4.965
8	-6.6	1.848	6.058
9	-6.6	8.342	12.114

Writing output ... done.

mode	affinity (kcal/mol)	dist from best mode rmsd l.b.	rmsd u.b.
1	-8.1	0.000	0.000
2	-8.1	0.593	5.123
3	-8.1	1.337	8.352
4	-8.0	0.636	4.698
5	-8.0	0.795	8.099
6	-7.9	1.561	6.599
7	-7.7	1.589	2.783
8	-7.6	1.342	7.674
9	-7.4	1.655	6.647

Writing output ... done.

mode	affinity (kcal/mol)	dist from best mode rmsd l.b.	rmsd u.b.
1	-4.7	0.000	0.000
2	-4.5	15.527	18.512
3	-4.4	3.999	7.354
4	-4.3	15.583	19.626
5	-4.3	15.843	18.726
6	-4.2	3.447	8.864
7	-4.2	15.687	19.621
8	-4.1	3.519	7.291
9	-4.1	15.672	18.924

Writing output ... done.

Figure S3. Docking affinity of CD4, CD5 and CD6 with the 6PVT model, respectively.

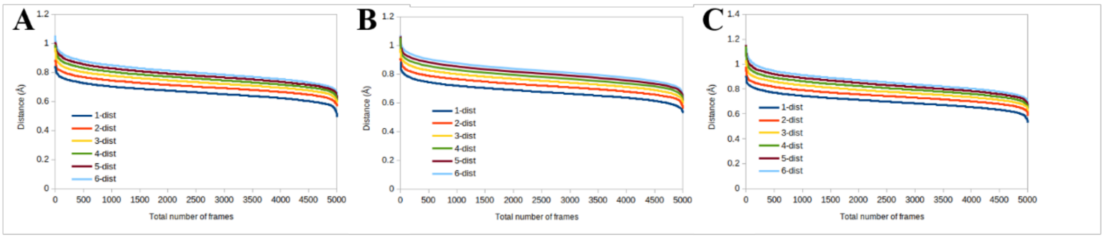

Figure S4. The K-dist of each system. (A)the 6PVT_CD4 system, (B)the 6PVT_CD5 system, and the 6PVT_CD6 system.

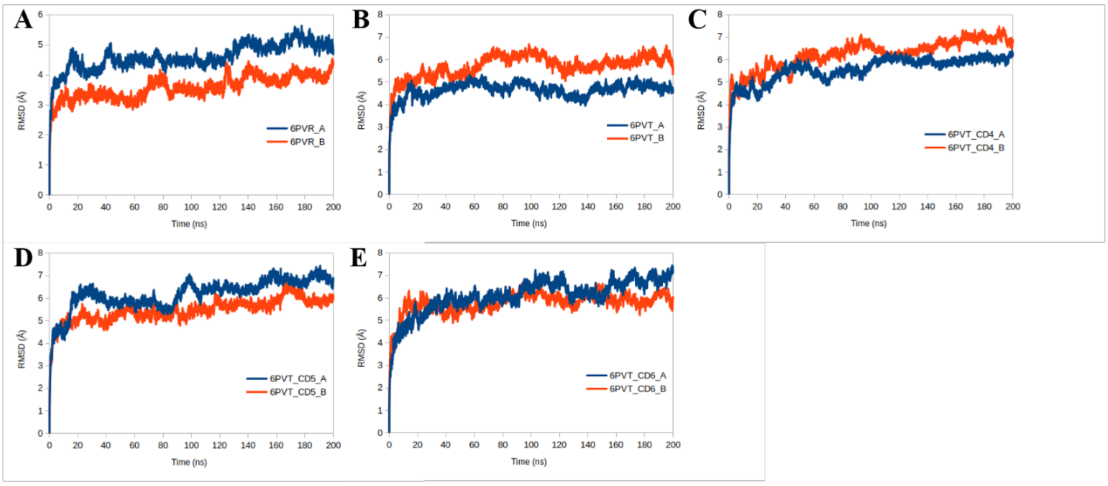

Figure S5. RMSD values of protein backbone atoms relative to the initial structure in the other two parallel molecular dynamics simulations, including the 6PVR and 6PVT archetypal protein models, as well as complex models with CDs (6PVT_CD4, 6PVT_CD5 and 6PVT_CD6).

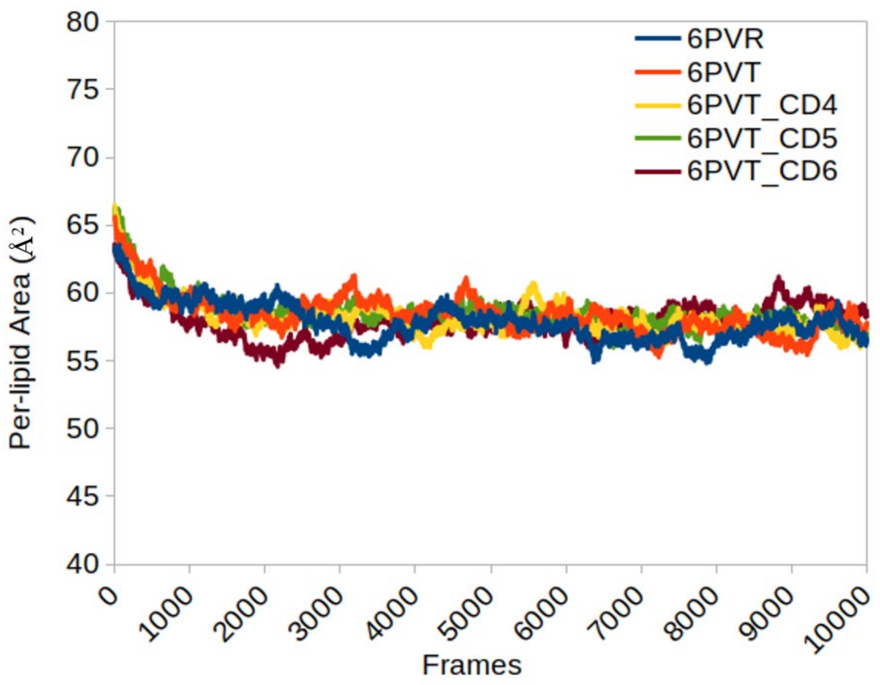

Figure S6. The per-lipids area of each frame.

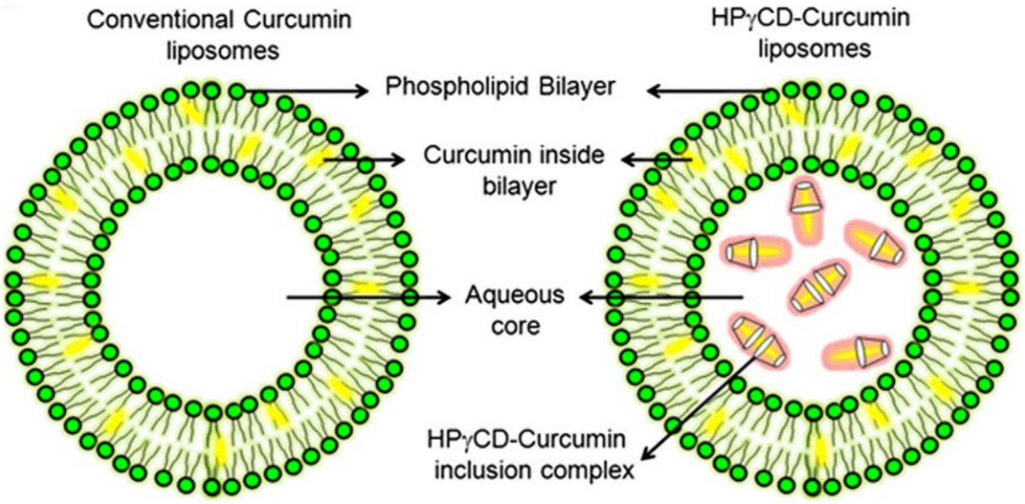

Figure S7. Schematic representation of conventional and HPγCD-curcumin liposomes. (refer in article [50])

Table S1. The number of lipids of each model.

Lipid Type	Upper-leaflet Number	Lower-leaflet Number
Cholesterol	16	17
POPC	48	51
POPG	16	17

Table S2. Binding Free Energy for the Three Complex Models: 6PVT_CD4, 6PVT_CD5, and 6PVT_CD6 (kcal/mol).

System	6PVT_CD4	6PVT_CD5	6PVT_CD6
$\Delta E_{vdw}{}^{1}$	-48.68	-53.18	-63.11
$\Delta E_{ele}{}^{2}$	-40.29	-72.77	-24.5
$\Delta G_{GB}{}^{3}$	51.87	94.72	55.08
$\Delta G_{SURF}{}^{4}$	-7.62	-7.76	-9.71
$\Delta G_{bind}{}^{5}$	-44.71	-38.99	-42.24

Note: $\Delta E_{vdw}{}^{1}$: van der Waals interaction energy; $\Delta E_{ele}{}^{2}$: electrostatic interaction energy; $\Delta G_{GB}{}^{3}$: polar solvation energy; $\Delta G_{SURF}{}^{4}$: non-polar solvation energy; $\Delta G_{bind}{}^{5}$: binding free energy.

Table S3. In the 6PVT_CD4 model, the residue decomposition energy of key residues (kcal/mol).

Residue	ΔE_{vdw}	ΔE_{ele}	ΔG_{GB}	ΔG_{SURF}	ΔG_{bind}
A_H19	-1.23	-10.71	8.22	-0.43	-4.15
C_S16	-1.63	-2.66	1.98	-0.28	-2.59
A_L15	-2.05	0.54	-0.67	-0.20	-2.36
D_S12	-1.23	-2.10	1.75	-0.24	-1.81
D_L15	-1.76	0.31	-0.10	-0.20	-1.74
C_L15	-1.55	0.30	-0.26	-0.15	-1.66
D_H19	-1.7	-1.44	1.82	-0.35	-1.66
A_S16	-1.42	-0.18	0.53	-0.12	-1.19
C_H19	-1.68	0.02	0.87	-0.37	-1.16
C_S12	-0.67	-1.48	1.18	-0.16	-1.13
D_S16	-1.53	-0.49	1.04	-0.13	-1.12

Table S4. In the 6PVT_CD5 model, the residue decomposition energy of key residues (kcal/mol).

Residue	ΔE_{vdw}	ΔE_{ele}	ΔG_{GB}	ΔG_{SURF}	ΔG_{bind}
C_ H19	-1.28	-14.21	12.61	-0.32	-3.20
D_L15	-1.99	0.32	-0.32	-0.27	-2.26

D_S12	-1.96	-2.12	2.44	-0.32	-1.97
C_L15	-1.46	0.53	-0.70	-0.13	-1.75
C_S16	-1.97	-0.53	1.49	-0.22	-1.23
A_F20	-1.35	-0.26	0.66	-0.2	-1.15
B_L15	-0.75	0.37	-0.67	-0.04	-1.10

Table S5. In the 6PVT_CD6 model, the residue decomposition energy of key residues (kcal/mol).

Residue	ΔE_{vdw}	ΔE_{ele}	ΔG_{GB}	ΔG_{SURF}	ΔG_{bind}
D_L15	-2.75	-0.44	0.42	-0.64	-3.42
A_S12	-3.11	-2.38	3.52	-0.48	-2.46
B_S9	-2.07	-0.66	0.80	-0.26	-2.19
A_S9	-1.71	-2.03	1.92	-0.24	-2.06
D_L8	-1.71	0.43	-0.05	-0.44	-1.77
B_S12	-1.78	-0.79	1.12	-0.21	-1.66
B_F13	-1.50	0.16	0.02	-0.08	-1.40
C_S12	-1.54	-0.64	1.06	-0.26	-1.37
C_L8	-1.13	-0.07	-0.04	-0.05	-1.29
A_L8	-1.12	0.11	0.15	-0.23	-1.09

Table S6. The hydrogen bond between CD4 and the residues of BM2 that occupancy more than 20%.

Acceptor	DonorH	Donor	Fraction
C_S16@OG	CD4@H29	CD4@O9	0.79
CD4@O6	A_H19@HD1	A_H19@ND1	0.64
CD4@O16	D_S12@HG	D_S12@OG	0.28
CD4@O18	B_H19@HD1	B_H19@ND1	0.21

Table S7. The hydrogen bond between CD5 and the residues of BM2 that occupancy more than

20%.

Acceptor	DonorH	Donor	Fraction
D_S12@OG	CD5@H25	CD5@O16	0.52
A_S12@OG	CD5@H29	CD5@O18	0.33
CD5@O20	C_H19@HD1	C_H19@ND1	0.22

Table S8. The hydrogen bond between CD6 and the residues of BM2 that occupancy more than 20%.

Acceptor	DonorH	Donor	Fraction
CD6@O59	A_S9@HG	A_S9@OG	0.33
CD6@O47	A_S9@HG	A_S9@OG	0.29
D_S12@OG	CD6@H58	CD6@O64	0.27
B_S9@O	CD6@H47	CD6@O53	0.21

Table S9. The occupancy of hydrogen bonds formed between residues Serine triplet and H19 with solvent molecules.

Residue	6PVR	6PVT	6PVT_CD4	6PVT_CD5	6PVT_CD6
A_S9@OG	0.12	0.49	0.75	0.69	0.01
B_S9@OG	None	0.20	0.80	0.68	0.31
C_S9@OG	0.43	0.81	0.72	0.54	0.38
D_S9@OG	0.17	0.42	0.80	0.58	None
A_S12@OG	0.45	0.72	0.65	0.34	0.72
B_S12@OG	0.70	0.48	0.38	0.67	0.58
C_S12@OG	0.37	0.28	0.65	0.69	None
D_S12@OG	0.60	0.69	0.06	0.24	0.01
A_S16@OG	0.41	0.73	0.37	0.33	0.65
B_S16@OG	0.58	0.39	0.76	0.88	0.52
C_S16@OG	0.83	0.46	0.87	0.01	0.28
D_S16@OG	0.14	0.36	0.65	0.58	0.30

A_H19@HD1	0.38	0.53	None	0.56	0.75
A_H19@HE2	0.08	0.67	0.28	0.47	0.45
B_H19@HD1	0.44	0.70	0.25	0.81	0.64
B_H19@HE2	0.47	0.57	0.47	0.59	0.62
C_H19@HD1	0.46	0.71	0.93	0.06	0.32
C_H19@HE2	0.16	0.62	0.62	0.46	0.45
D_H19@HD1	0.23	0.71	0.77	0.10	0.52
D_H19@HE2	0.01	None	0.34	0.23	0.14

Note: Green indicates data showing a decrease in hydrogen bond occupancy compared to the empty protein model 6PVT, while gray indicates data showing hydrogen bond occupancy remaining relatively consistent compared to the empty protein model 6PVT.

Table S10. The centers and sizes of docking boxes in x, y, and z dimensions (Å).

ligand	CD4	CD5	CD6
center_x	39.829	36.922	36.922
center_y	10.164	10.164	9.357
center_z	16.521	9.772	11.44
size_x	37.59	39	38.25
size_y	32.06	39	23.25
size_z	38.69	41.25	36

Table S11. The water molecules number of each system.

Residue	6PVR	6PVT	6PVT_CD4	6PVT_CD5	6PVT_CD6
#water	8245	8269	8259	8255	8251

Table S12. The model size of each system.

Model	X（Å）	Y（Å）	Z（Å）
6PVR	76.80	76.80	82.30
6PVT	75.70	75.70	85.30
6PVT_CD4	76.70	76.70	85.30

6PVT_CD5	76.80	76.80	85.30
6PVT_CD6	76.90	76.90	85.30

Table S13. Parameters of lipid solubility and water solubility of CD4, CD5, and CD6.

CDs	LogS, Log of the aqueous solubility. Optimal: -4~0.5 (log mol/L)	LogD, logP at physiological pH 7.4. Optimal: 1~3 (log mol/L)	LogP, Log of the octanol/water partition coefficient. Optimal: 0~3 (log mol/L)	TPSA, Topological Polar Surface Area. Optimal:0~140 (Å^2)
CD4	-1.775	-4.323	-2.858	316.6
CD5	-1.655	-5.064	-3.214	395.75
CD6	-1.587	-5.805	-3.651	474.9

Note: the below explanation comes from https://admetmesh.scbdd.com/explanation/index.
TPSA: Topological polar surface area. Sum of tabulated surface contributions of polar fragments. Optimal:0~140, based on Veber rule.
LogS: The logarithm of aqueous solubility value. The first step in the drug absorption process is the disintegration of the tablet or capsule, followed by the dissolution of the active drug. Low solubility is detrimental to good and complete oral absorption, and early measurement of this property is of great importance in drug discovery.
LogP: The logarithm of the n-octanol/water distribution coefficient. log P possess a leading position with considerable impact on both membrane permeability and hydrophobic binding to macromolecules, including the target receptor as well as other proteins like plasma proteins, transporters, or metabolizing enzymes.
logD7.4: The logarithm of the n-octanol/water distribution coefficients at pH=7.4. To exert a therapeutic effect, one drug must enter the blood circulation and then reach the site of action. Thus, an eligible drug usually needs to keep a balance between lipophilicity and hydrophilicity to dissolve in the body fluid and penetrate the biomembrane effectively. Therefore, it is important to estimate the n-octanol/water distribution coefficients at physiological pH (logD7.4) values for candidate compounds in the early stage of drug discovery.

The partial charges of each atom of each molecule.

CD4

Atom	x	y	z	name	partial charge
1 C1	-3.2310	1.3350	3.4170 c2	1 AGL	0.118000
2 H1	-4.3330	1.2600	3.5430 h4	1 AGL	0.136000
3 C5	-1.1250	1.6290	4.6560 c3	1 AGL	-0.026000
4 H5	-0.7720	0.6300	4.3220 h1	1 AGL	0.113000
5 O5	-2.6120	1.6420	4.7020 os	1 AGL	-0.292000

6 C2	-2.8910	2.4600	2.4310 c3	1 AGL	-0.009000
7 H2	-3.3070	3.4180	2.8080 h1	1 AGL	0.129000
8 O2	-3.4450	2.2040	1.1160 oh	1 AGL	-0.306000
9 HO2	-3.6370	1.2670	1.0350 ho	1 AGL	0.227000
10 C3	-1.3660	2.5700	2.3230 c3	1 AGL	-0.008000
11 H3	-0.9610	1.6420	1.8660 h1	1 AGL	0.152000
12 O3	-0.9660	3.7030	1.4980 oh	1 AGL	-0.309000
13 HO3	-1.3050	4.5110	1.8910 ho	1 AGL	0.203000
14 C4	-0.6550	2.7100	3.6670 c3	1 AGL	-0.001000
15 H4	-0.8710	3.7130	4.0920 h1	1 AGL	0.097000
16 O4	0.7410	2.4370	3.3540 os	1 AGL	-0.373800
17 C6	-0.6090	1.7400	6.0970 c3	1 AGL	-0.027000
18 H61	-1.4240	2.0900	6.7660 h1	1 AGL	0.103000
19 H62	0.2390	2.4560	6.1420 h1	1 AGL	0.103000
20 O6	-0.1530	0.4350	6.5460 oh	1 AGL	-0.325000
21 HO6	0.8040	0.4480	6.6240 ho	1 AGL	0.219000
22 C1	1.8450	3.3170	3.6320 c3	2 AGL	0.306900
23 H1	1.4540	4.3120	3.9320 h2	2 AGL	0.096700
24 C5	2.9570	1.3480	4.5240 c3	2 AGL	0.084100
25 H5	2.0090	0.7760	4.6150 h1	2 AGL	0.075700
26 O5	2.6550	2.7830	4.6970 os	2 AGL	-0.454600
27 C2	2.6830	3.3300	2.3590 c3	2 AGL	0.104100
28 H2	3.6170	3.9020	2.5450 h1	2 AGL	0.090700
29 O2	1.9960	3.9390	1.2400 oh	2 AGL	-0.576800
30 HO2	1.0510	3.7970	1.3390 ho	2 AGL	0.428000
31 C3	3.0580	1.8870	1.9980 c3	2 AGL	0.104100
32 H3	2.1530	1.3420	1.6550 h1	2 AGL	0.112700
33 O3	4.0480	1.8610	0.9360 oh	2 AGL	-0.579800
34 HO3	4.8840	2.1820	1.2800 ho	2 AGL	0.403000
35 C4	3.6070	1.0310	3.1510 c3	2 AGL	0.115100
36 H4	4.7010	1.2040	3.2300 h1	2 AGL	0.058700
37 O4	3.2260	-0.3260	2.7130 os	2 AGL	-0.448600
38 C6	3.8090	0.9850	5.7240 c3	2 AGL	0.122400
39 H61	4.5250	1.8010	5.9570 h1	2 AGL	0.067700
40 H62	4.3690	0.0440	5.5350 h1	2 AGL	0.067700
41 O6	2.8910	0.7950	6.8520 oh	2 AGL	-0.598800
42 HO6	3.3830	0.8750	7.6720 ho	2 AGL	0.414000
43 C1	3.7500	-1.5560	3.2490 c3	3 AGL	0.305900
44 H1	4.8450	-1.4370	3.3940 h2	3 AGL	0.096700
45 C5	1.6790	-2.0300	4.5360 c3	3 AGL	0.095100
46 H5	1.2490	-1.0160	4.3870 h1	3 AGL	0.064700
47 O5	3.1640	-1.9340	4.5070 os	3 AGL	-0.439600
48 C2	3.3790	-2.5930	2.1850 c3	3 AGL	0.102100
49 H2	3.7800	-3.5850	2.4840 h1	3 AGL	0.093700

50	O2	3.9180	-2.2560	0.8800	oh	3 AGL	-0.576800
51	HO2	4.1290	-1.3200	0.8610	ho	3 AGL	0.425000
52	C3	1.8550	-2.6770	2.0940	c3	3 AGL	0.095100
53	H3	1.4460	-1.7000	1.7580	h1	3 AGL	0.111700
54	O3	1.4560	-3.7050	1.1410	oh	3 AGL	-0.587800
55	HO3	1.6630	-4.5680	1.5060	ho	3 AGL	0.407000
56	C4	1.1640	-2.9720	3.4260	c3	3 AGL	0.110100
57	H4	1.3660	-4.0250	3.7180	h1	3 AGL	0.077700
58	O4	-0.2340	-2.6410	3.1950	os	3 AGL	-0.456600
59	C6	1.3440	-2.4200	5.9840	c3	3 AGL	0.129400
60	H61	1.8700	-1.7300	6.6770	h1	3 AGL	0.061200
61	H62	1.6850	-3.4610	6.1680	h1	3 AGL	0.061200
62	O6	-0.0640	-2.3530	6.2670	oh	3 AGL	-0.608800
63	HO6	-0.3060	-1.4380	6.4310	ho	3 AGL	0.429000
64	C1	-1.3420	-3.5680	3.2310	c3	4 AGL	0.303900
65	H1	-0.9530	-4.5950	3.3990	h2	4 AGL	0.111700
66	C5	-2.5080	-1.7780	4.3950	c3	4 AGL	0.085100
67	H5	-1.5600	-1.2580	4.6520	h1	4 AGL	0.070700
68	O5	-2.2530	-3.2300	4.2880	os	4 AGL	-0.425600
69	C2	-2.1000	-3.3740	1.9190	c3	4 AGL	0.094100
70	H2	-3.0450	-3.9570	1.9660	h1	4 AGL	0.089700
71	O2	-1.3790	-3.7990	0.7410	oh	4 AGL	-0.581800
72	HO2	-0.4380	-3.6900	0.8940	ho	4 AGL	0.430000
73	C3	-2.4320	-1.8850	1.7960	c3	4 AGL	0.111100
74	H3	-1.4980	-1.3090	1.6250	h1	4 AGL	0.109700
75	O3	-3.3390	-1.6370	0.6840	oh	4 AGL	-0.581800
76	HO3	-4.2340	-1.8490	0.9570	ho	4 AGL	0.402000
77	C4	-3.0540	-1.2380	3.0420	c3	4 AGL	0.198800
78	H4	-4.1500	-1.4110	3.0140	h1	4 AGL	0.055700
79	O4	-2.6690	0.1780	2.8110	o	4 AGL	-0.465500
80	C6	-3.4410	-1.6450	5.6100	c3	4 AGL	0.138400
81	H61	-3.6400	-2.6580	6.0210	h1	4 AGL	0.055200
82	H62	-4.3970	-1.1860	5.2790	h1	4 AGL	0.055200
83	O6	-2.9040	-0.8300	6.6790	oh	4 AGL	-0.598800
84	HO6	-3.5410	-0.7960	7.3960	ho	4 AGL	0.427000

CD5

1	C1	-0.4840	4.8990	3.8230	c2	1 4GA	0.123000
2	H1	-1.2460	5.6720	3.9240	h4	1 4GA	0.139000
3	C2	0.5280	5.2600	2.7530	c3	1 4GA	-0.021000
4	H2	1.0130	6.1890	3.0570	h1	1 4GA	0.139000
5	O2	-0.1650	5.4790	1.5220	oh	1 4GA	-0.325000
6	H2O	-0.8630	4.8160	1.4290	ho	1 4GA	0.233000

7	C3	1.5510	4.1500	2.5950	c3	1 4GA	-0.007000
8	H3	1.0580	3.2600	2.1980	h1	1 4GA	0.110000
9	O3	2.5280	4.6120	1.6530	oh	1 4GA	-0.338000
10	H3O	2.0510	5.0420	0.9300	ho	1 4GA	0.225000
11	C4	2.2230	3.8850	3.9170	c3	1 4GA	0.023000
12	H4	2.8160	4.7550	4.2040	h1	1 4GA	0.112000
13	C5	1.1730	3.5950	4.9760	c3	1 4GA	-0.021000
14	H5	0.7120	2.6320	4.7470	h1	1 4GA	0.115000
15	C6	1.7800	3.5290	6.3740	c3	1 4GA	-0.018000
16	H62	2.1890	2.5380	6.5700	h1	1 4GA	0.105500
17	H61	2.5930	4.2520	6.4630	h1	1 4GA	0.105500
18	O6	0.8260	3.7990	7.3990	oh	1 4GA	-0.340000
19	H6O	0.2830	3.0220	7.5500	ho	1 4GA	0.232000
20	O5	0.2400	4.7020	5.0390	os	1 4GA	-0.283000
21	O4	2.9370	2.6320	3.7460	os	1 4GA	-0.381800
22	C1	4.3480	2.4590	3.7570	c3	2 4GA	0.305900
23	H1	4.8410	3.4280	3.8430	h2	2 4GA	0.099700
24	C2	4.7400	1.7820	2.4440	c3	2 4GA	0.090100
25	H2	5.8190	1.6260	2.4620	h1	2 4GA	0.099700
26	O2	4.4320	2.6250	1.3320	oh	2 4GA	-0.597800
27	H2O	3.5790	3.0540	1.4850	ho	2 4GA	0.434000
28	C3	4.0200	0.4520	2.3210	c3	2 4GA	0.104100
29	H3	2.9470	0.6310	2.2290	h1	2 4GA	0.070700
30	O3	4.4850	-0.1980	1.1290	oh	2 4GA	-0.610800
31	H3O	4.5280	0.4710	0.4320	ho	2 4GA	0.426000
32	C4	4.3720	-0.4090	3.4980	c3	2 4GA	0.134100
33	H4	5.4310	-0.6740	3.4510	h1	2 4GA	0.072700
34	C5	4.0880	0.3110	4.8000	c3	2 4GA	0.090100
35	H5	3.0110	0.2810	4.9790	h1	2 4GA	0.075700
36	C6	4.8210	-0.3790	5.9440	c3	2 4GA	0.132400
37	H62	5.7870	-0.7570	5.6100	h1	2 4GA	0.066200
38	H61	4.9980	0.3290	6.7550	h1	2 4GA	0.066200
39	O6	4.1320	-1.4930	6.5000	oh	2 4GA	-0.612800
40	H6O	4.2380	-2.2570	5.9290	ho	2 4GA	0.433000
41	O5	4.7670	1.5900	4.8270	os	2 4GA	-0.426600
42	O4	3.2890	-1.3800	3.4140	os	2 4GA	-0.453600
43	C1	3.4710	-2.7750	3.5150	c3	3 4GA	0.305900
44	H1	4.5280	-3.0040	3.6490	h2	3 4GA	0.099700
45	C2	2.9610	-3.4180	2.2270	c3	3 4GA	0.090100
46	H2	3.0980	-4.4970	2.3210	h1	3 4GA	0.099700
47	O2	3.7250	-2.9690	1.1030	oh	3 4GA	-0.597800
48	H2O	3.9060	-2.0240	1.1990	ho	3 4GA	0.434000
49	C3	1.4730	-3.1370	2.0810	c3	3 4GA	0.104100
50	H3	1.3210	-2.0630	1.9540	h1	3 4GA	0.070700

51	O3	0.9650	-3.7990	0.9150	oh	3 4GA	-0.610800
52	H3O	1.6080	-3.6660	0.2050	ho	3 4GA	0.426000
53	C4	0.7270	-3.6620	3.2840	c3	3 4GA	0.134100
54	H4	0.7940	-4.7520	3.3060	h1	3 4GA	0.072700
55	C5	1.3110	-3.0860	4.5600	c3	3 4GA	0.090100
56	H5	1.0030	-2.0410	4.6360	h1	3 4GA	0.075700
57	C6	0.8140	-3.8410	5.7980	c3	3 4GA	0.132400
58	H62	0.7290	-4.9070	5.5900	h1	3 4GA	0.066200
59	H61	1.5180	-3.7130	6.6210	h1	3 4GA	0.066200
60	O6	-0.4770	-3.4160	6.2640	oh	3 4GA	-0.612800
61	H6O	-1.1630	-3.7990	5.7120	ho	3 4GA	0.433000
62	O5	2.7290	-3.3640	4.6050	os	3 4GA	-0.426600
63	O4	-0.5410	-2.9900	3.0880	os	3 4GA	-0.453600
64	C1	-1.8080	-3.5860	3.2070	c3	4 4GA	0.305900
65	H1	-1.7020	-4.6650	3.3260	h2	4 4GA	0.099700
66	C2	-2.6010	-3.2820	1.9450	c3	4 4GA	0.090100
67	H2	-3.5540	-3.8070	2.0190	h1	4 4GA	0.099700
68	O2	-1.8930	-3.7790	0.8000	oh	4 4GA	-0.597800
69	H2O	-0.9480	-3.6050	0.9110	ho	4 4GA	0.434000
70	C3	-2.8040	-1.7890	1.8090	c3	4 4GA	0.104100
71	H3	-1.8370	-1.3080	1.6440	h1	4 4GA	0.070700
72	O3	-3.6340	-1.5250	0.6780	oh	4 4GA	-0.610800
73	H3O	-3.3330	-2.0960	-0.0410	ho	4 4GA	0.426000
74	C4	-3.4850	-1.2120	3.0220	c3	4 4GA	0.134100
75	H4	-4.4980	-1.6130	3.0960	h1	4 4GA	0.072700
76	C5	-2.6630	-1.5830	4.2500	c3	4 4GA	0.090100
77	H5	-1.7060	-1.0600	4.1920	h1	4 4GA	0.075700
78	C6	-3.2840	-1.2220	5.5810	c3	4 4GA	0.132400
79	H62	-3.8160	-0.2730	5.5130	h1	4 4GA	0.066200
80	H61	-4.0010	-1.9890	5.8790	h1	4 4GA	0.066200
81	O6	-2.2870	-1.0950	6.6010	oh	4 4GA	-0.612800
82	H6O	-1.8450	-0.2470	6.5180	ho	4 4GA	0.433000
83	O5	-2.5220	-3.0270	4.3200	os	4 4GA	-0.426600
84	O4	-3.3300	0.1990	2.7680	os	4 4GA	-0.453600
85	C1	-4.2810	1.1630	3.1220	c3	5 4GA	0.305900
86	H1	-5.2770	0.7210	3.1230	h2	5 4GA	0.099700
87	C2	-4.1790	2.2650	2.0750	c3	5 4GA	0.090100
88	H2	-4.9390	3.0120	2.3070	h1	5 4GA	0.099700
89	O2	-4.4540	1.7160	0.7760	oh	5 4GA	-0.597800
90	H2O	-4.0370	0.8460	0.7040	ho	5 4GA	0.434000
91	C3	-2.8010	2.8950	2.1090	c3	5 4GA	0.104100
92	H3	-2.0630	2.1660	1.7680	h1	5 4GA	0.070700
93	O3	-2.7630	4.0250	1.2130	oh	5 4GA	-0.610800
94	H3O	-3.2150	3.7630	0.3990	ho	5 4GA	0.426000

95 C4	-2.4750	3.4050	3.4920 c3	5 4GA	0.223800
96 H4	-3.1480	4.2260	3.7450 h1	5 4GA	0.072700
97 C5	-2.6280	2.2620	4.4900 c3	5 4GA	0.090100
98 H5	-1.8950	1.4910	4.2420 h1	5 4GA	0.075700
99 C6	-2.4070	2.6290	5.9450 c3	5 4GA	0.132400
100 H62	-2.0910	3.6680	6.0360 h1	5 4GA	0.066200
101 H61	-3.3360	2.5090	6.5050 h1	5 4GA	0.066200
102 O6	-1.4010	1.8210	6.5530 oh	5 4GA	-0.612800
103 H6O	-0.5320	2.1340	6.2920 ho	5 4GA	0.433000
104 O5	-3.9740	1.7420	4.4010 os	5 4GA	-0.426600
105 O4	-1.0730	3.6960	3.3840 o	5 4GA	-0.471500

CD6

1 C1	33.0040	9.8300	6.2750 c3	1 GLC	0.305067
2 O1	30.9260	9.6860	5.0720 oh	1 GLC	-0.587467
3 C2	31.9890	8.8910	5.6090 c3	1 GLC	0.101267
4 O2	30.7160	6.8730	5.9410 oh	1 GLC	-0.588967
5 C3	31.3840	7.9250	6.6280 c3	1 GLC	0.099267
6 O3	31.6510	6.8610	8.7320 os	1 GLC	-0.446100
7 C4	32.4060	7.3590	7.6290 c3	1 GLC	0.104767
8 O4	33.9790	9.1610	7.0750 os	1 GLC	-0.430933
9 C5	33.4970	8.3200	8.1190 c3	1 GLC	0.092100
10 O5	35.0460	8.4170	9.9510 oh	1 GLC	-0.604800
11 C6	34.6320	7.6160	8.8610 c3	1 GLC	0.128567
12 H01	33.5060	10.3420	5.4540 h2	1 GLC	0.096033
13 H02	32.4990	8.3190	4.8340 h1	1 GLC	0.086867
14 H04	30.6750	8.4960	7.2270 h1	1 GLC	0.096867
15 H06	32.9780	6.5940	7.1050 h1	1 GLC	0.065200
16 H07	33.0160	8.9650	8.8550 h1	1 GLC	0.077867
17 H08	35.4710	7.4620	8.1820 h1	1 GLC	0.069283
18 H09	34.2840	6.6510	9.2290 h1	1 GLC	0.069283
19 H03	30.8680	10.5130	5.5570 ho	1 GLC	0.432000
20 H05	29.8800	7.1950	5.5970 ho	1 GLC	0.411833
21 H10	35.2650	7.8540	10.6980 ho	1 GLC	0.421500
22 O6	29.6410	5.0670	8.1350 oh	2 GLC	-0.587467
23 C7	31.8690	5.4610	8.9300 c3	2 GLC	0.305067
24 O7	28.6680	4.8470	10.8300 oh	2 GLC	-0.588967
25 C8	30.5220	4.8190	9.2330 c3	2 GLC	0.101267
26 O8	30.4770	6.2350	12.6550 os	2 GLC	-0.446100
27 C9	29.9580	5.4080	10.5300 c3	2 GLC	0.099267
28 O9	32.8050	5.2340	9.9700 os	2 GLC	-0.430933
29 C10	30.9500	5.2750	11.7090 c3	2 GLC	0.104767
30 O10	33.6850	6.1470	13.2620 oh	2 GLC	-0.604800

31 C11	32.4130	5.5950	11.2950 c3	2 GLC	0.092100
32 C12	33.5460	5.1890	12.2420 c3	2 GLC	0.128567
33 H01	30.6320	3.7430	9.3650 h1	2 GLC	0.086867
34 H02	29.8180	6.4780	10.3750 h1	2 GLC	0.096867
35 H03	30.9830	4.2570	12.0960 h1	2 GLC	0.065200
36 H04	32.3040	6.6780	11.3590 h1	2 GLC	0.077867
37 H05	34.4780	5.1270	11.6800 h1	2 GLC	0.069283
38 H06	33.3180	4.2190	12.6850 h1	2 GLC	0.069283
39 H10	32.2950	5.0130	8.0320 h2	2 GLC	0.096033
40 H12	30.0750	4.8230	7.3140 ho	2 GLC	0.432000
41 H14	28.7700	4.1250	11.4550 ho	2 GLC	0.411833
42 H19	33.7450	7.0230	12.8720 ho	2 GLC	0.421500
43 O11	27.9850	5.5210	13.5090 oh	3 GLC	-0.587467
44 O12	27.2830	7.8560	15.0900 oh	3 GLC	-0.588967
45 C13	30.3100	5.8260	14.0190 c3	3 GLC	0.305067
46 O13	29.5910	9.7530	14.9330 os	3 GLC	-0.446100
47 C14	28.8680	6.0940	14.4780 c3	3 GLC	0.101267
48 O14	31.2990	6.5470	14.7590 os	3 GLC	-0.430933
49 C15	28.5850	7.6000	14.5650 c3	3 GLC	0.099267
50 O15	31.8850	7.7360	17.3630 oh	3 GLC	-0.604800
51 C16	29.6640	8.3780	15.3450 c3	3 GLC	0.104767
52 C17	31.1200	7.9290	15.0910 c3	3 GLC	0.092100
53 C18	32.1600	8.3660	16.1250 c3	3 GLC	0.128567
54 H01	28.7210	5.6570	15.4660 h1	3 GLC	0.086867
55 H02	28.6200	7.9730	13.5410 h1	3 GLC	0.096867
56 H03	29.4520	8.2000	16.3990 h1	3 GLC	0.065200
57 H04	31.3250	8.5050	14.1890 h1	3 GLC	0.077867
58 H05	32.1170	9.4480	16.2500 h1	3 GLC	0.069283
59 H06	33.1550	8.0810	15.7840 h1	3 GLC	0.069283
60 H07	30.4550	4.7560	14.1690 h2	3 GLC	0.096033
61 H22	27.1070	5.8940	13.6160 ho	3 GLC	0.432000
62 H24	26.8360	7.0240	15.2600 ho	3 GLC	0.411833
63 H29	31.2910	8.2870	17.8780 ho	3 GLC	0.421500
64 O16	27.1280	10.8780	15.7990 oh	4 GLC	-0.587467
65 O17	27.4700	13.3040	14.3830 oh	4 GLC	-0.588967
66 O18	30.0580	13.5860	13.2350 os	4 GLC	-0.446100
67 C19	29.5260	10.6510	16.0560 c3	4 GLC	0.305067
68 O19	30.7370	11.4110	16.1410 os	4 GLC	-0.430933
69 C20	28.3470	11.6140	15.8670 c3	4 GLC	0.101267
70 O20	33.0080	12.5790	13.7010 oh	4 GLC	-0.604800
71 C21	28.5360	12.3690	14.5610 c3	4 GLC	0.099267
72 C22	29.8980	13.0860	14.5630 c3	4 GLC	0.104767
73 C23	31.0100	12.0670	14.8900 c3	4 GLC	0.092100
74 C24	32.3870	12.7120	14.9650 c3	4 GLC	0.128567

75	H01	28.3060	12.3080	16.7070	h1	4 GLC	0.086867
76	H02	28.5190	11.6640	13.7300	h1	4 GLC	0.096867
77	H03	29.9520	13.8840	15.3030	h1	4 GLC	0.065200
78	H04	31.0170	11.3430	14.0750	h1	4 GLC	0.077867
79	H05	32.9890	12.2250	15.7320	h1	4 GLC	0.069283
80	H06	32.2870	13.7670	15.2190	h1	4 GLC	0.069283
81	H07	29.3940	10.0720	16.9700	h2	4 GLC	0.096033
82	H32	27.2850	9.9730	16.0780	ho	4 GLC	0.432000
83	H34	27.6970	14.1340	14.8100	ho	4 GLC	0.411833
84	H39	33.0520	13.4380	13.2730	ho	4 GLC	0.421500
85	O21	28.0610	15.2560	12.4140	oh	5 GLC	-0.587467
86	O22	28.7940	15.5110	9.7380	oh	5 GLC	-0.588967
87	O23	31.4980	14.1220	9.2610	os	5 GLC	-0.446100
88	O24	31.7180	15.1600	12.7710	os	5 GLC	-0.430933
89	C25	30.3430	14.9820	13.1180	c3	5 GLC	0.305067
90	O25	34.3350	14.3680	10.7010	oh	5 GLC	-0.604800
91	C26	29.4120	15.5440	12.0480	c3	5 GLC	0.101267
92	C27	29.6820	14.9040	10.6840	c3	5 GLC	0.099267
93	C28	31.1550	15.0750	10.2720	c3	5 GLC	0.104767
94	C29	32.1360	14.8120	11.4350	c3	5 GLC	0.092100
95	C30	33.5160	15.4140	11.1830	c3	5 GLC	0.128567
96	H01	29.5850	16.6180	11.9760	h1	5 GLC	0.086867
97	H02	29.5010	13.8300	10.7260	h1	5 GLC	0.096867
98	H03	31.2440	16.1060	9.9300	h1	5 GLC	0.065200
99	H04	32.1650	13.7220	11.4240	h1	5 GLC	0.077867
100	H05	33.9280	15.8440	12.0960	h1	5 GLC	0.069283
101	H06	33.4490	16.2110	10.4430	h1	5 GLC	0.069283
102	H07	30.1770	15.5090	14.0580	h2	5 GLC	0.096033
103	H42	28.0260	15.0110	13.3420	ho	5 GLC	0.432000
104	H44	28.8630	15.0560	8.8960	ho	5 GLC	0.411833
105	H49	35.2580	14.6010	10.8250	ho	5 GLC	0.421500
106	O26	29.7000	14.7200	7.2580	oh	6 GLC	-0.587467
107	O27	30.1540	12.2070	5.8650	oh	6 GLC	-0.588967
108	O28	32.3120	10.7040	7.1760	os	6 GLC	-0.446100
109	O29	33.3190	14.1220	7.8210	os	6 GLC	-0.430933
110	O30	34.9300	11.2550	8.9650	oh	6 GLC	-0.604800
111	C31	32.0020	14.6600	8.0300	c3	6 GLC	0.305067
112	C32	31.0000	14.2360	6.9380	c3	6 GLC	0.101267
113	C33	30.9560	12.7050	6.9290	c3	6 GLC	0.099267
114	C34	32.3740	12.0930	6.8600	c3	6 GLC	0.104767
115	C35	33.3080	12.7030	7.9300	c3	6 GLC	0.092100
116	C36	34.7220	12.1190	7.8700	c3	6 GLC	0.128567
117	H01	31.3040	14.6370	5.9710	h1	6 GLC	0.086867
118	H02	30.4990	12.4010	7.8710	h1	6 GLC	0.096867

119 H03	32.7480	12.2860	5.8540 h1		6 GLC	0.065200
120 H04	32.9120	12.4350	8.9090 h1		6 GLC	0.077867
121 H05	35.4480	12.9310	7.9100 h1		6 GLC	0.069283
122 H06	34.8450	11.5630	6.9410 h1		6 GLC	0.069283
123 H07	32.0930	15.7460	8.0260 h2		6 GLC	0.096033
124 H52	29.2450	14.9750	6.4520 ho		6 GLC	0.432000
125 H54	29.8300	12.9410	5.3380 ho		6 GLC	0.411833
126 H59	34.9970	10.3500	8.6500 ho		6 GLC	0.421500
